# Supplementary material for: mRNA Deadenylation Is Coupled to Translation Rates by the Differential Activities of Ccr4-Not Nucleases
Source: Mol Cell. 2018 Jun 21;70(6):1089–1100.e8. doi: 10.1016/j.molcel.2018.05.033 (PMC6024076; doi:10.1016/j.molcel.2018.05.033)
Supplement: Document S1. Figures S1–S7 and Table S1 [file mmc1.pdf]

Molecular Cell, Volume *70*

Supplemental Information

mRNA Deadenylation Is Coupled to Translation Rates by the Differential Activities of Ccr4-Not Nucleases

Michael W. Webster, Ying-Hsin Chen, James A.W. Stowell, Najwa Alhusaini, Thomas Sweet, Brenton R. Graveley, Jeff Coller, and Lori A. Passmore

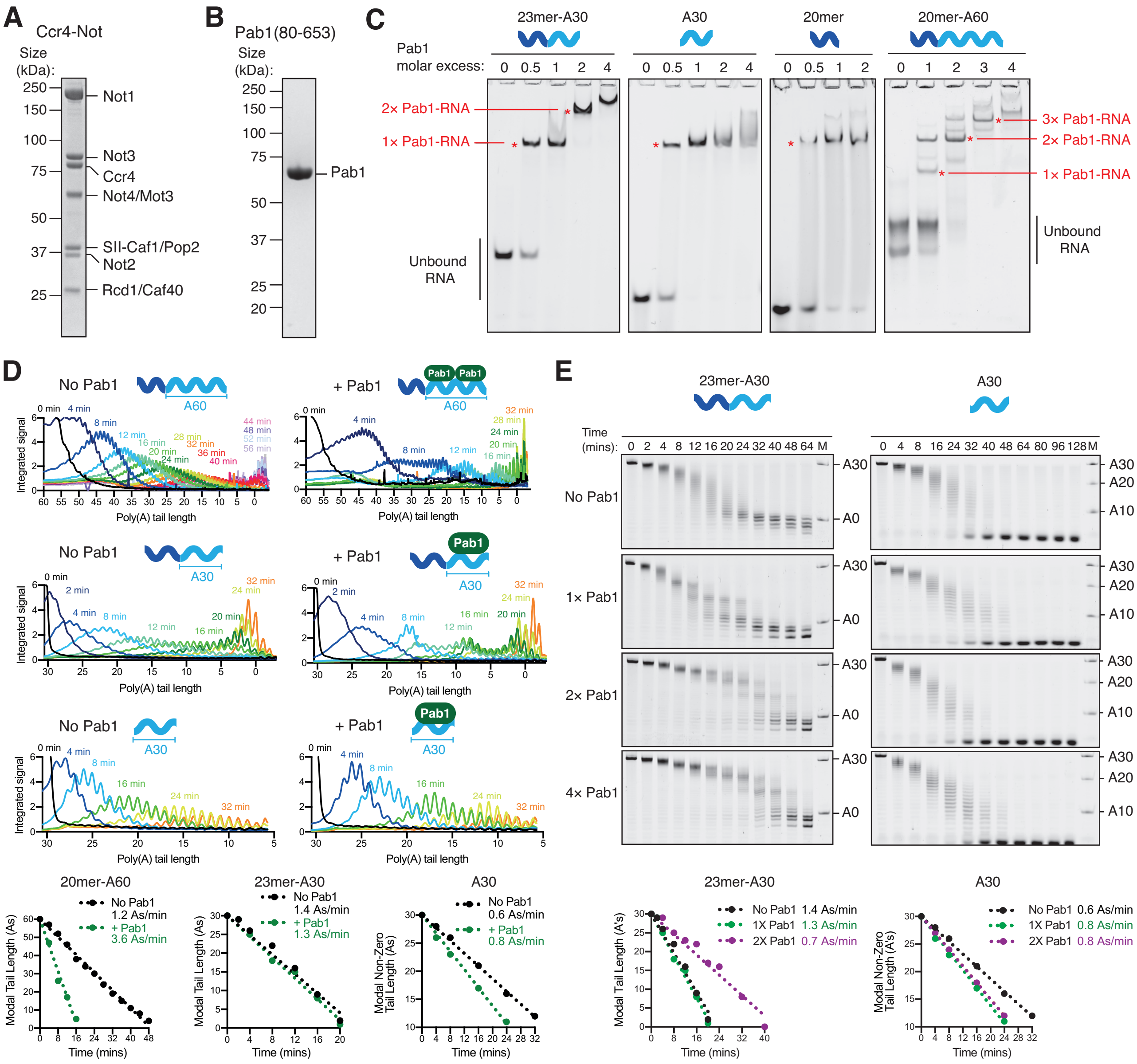

Figure S1. Reconstitution and analysis of deadenylation on Pab1-bound RNAs, Related to Figure 1.

(**A**) Coomassie-stained SDS-PAGE of *S. pombe* Ccr4-Not complex purified after overexpression in *Sf*9 insect cells. (**B**) Coomassie stained SDS-PAGE of *S. pombe* Pab1 (residues 80-653) purified following overexpression in *E. coli*. (**C**) Electrophoretic mobility shift assays (EMSAs) showing the Pab1-RNA complexes used as substrates for deadenylation assays. Purified Pab1 was mixed with RNA in the indicated molar ratio relative to RNA, and resolved on a 6% non-denaturing polyacrylamide gel. Gels containing 23mer-A30, A30, 20mer and 20mer-A60 were imaged to detect the 5′ fluorescent label. Gel containing 20mer-A60 was stained with SYBR Green II before scanning for fluorescence. Notably, Pab1 also binds to the non-poly(A) 20mer RNA. (**D**) Densitometric analyses of gels (top) and plots of the most abundant RNA poly(A) tail

length versus time (bottom) (Webster et al., 2017) for assays with 20mer-A60, 23mer-A30, and A30. Linear regression was applied to obtain the indicated reaction rates. 95% confidence intervals: ±0.04 (20mer-A60 no Pab1), ±0.6 (20mer-A60 + Pab1), ±0.1 (23mer-A30 no Pab1), ±0.1 (23mer-A30 + Pab1), ±0.02 (A30 no Pab1), ±0.05 (A30 + Pab1). (**E**) Deadenylation reactions performed with Ccr4-Not and a series of Pab1 concentrations. Pab1 was mixed with RNA in the indicated molar ratio relative to RNA to form the complexes indicated in (C). Experiments were performed with 23mer-A30 (left) and A30 (right), and poly(A) tail lengths are indicated. Reaction rates were determined as in (D). 95% confidence intervals: ±0.07 (23mer-A30 2X Pab1), ±0.05 (A30 2X Pab1). Interestingly, in our deadenylation assays using an A30 substrate lacking any upstream non-poly(A) sequence, Ccr4-Not did not proceed in step-wise manner (Figures S1D and S1E). Therefore, step-wise deadenylation likely occurs because the 3′-UTR stabilizes and positions Pab1 on RNA.

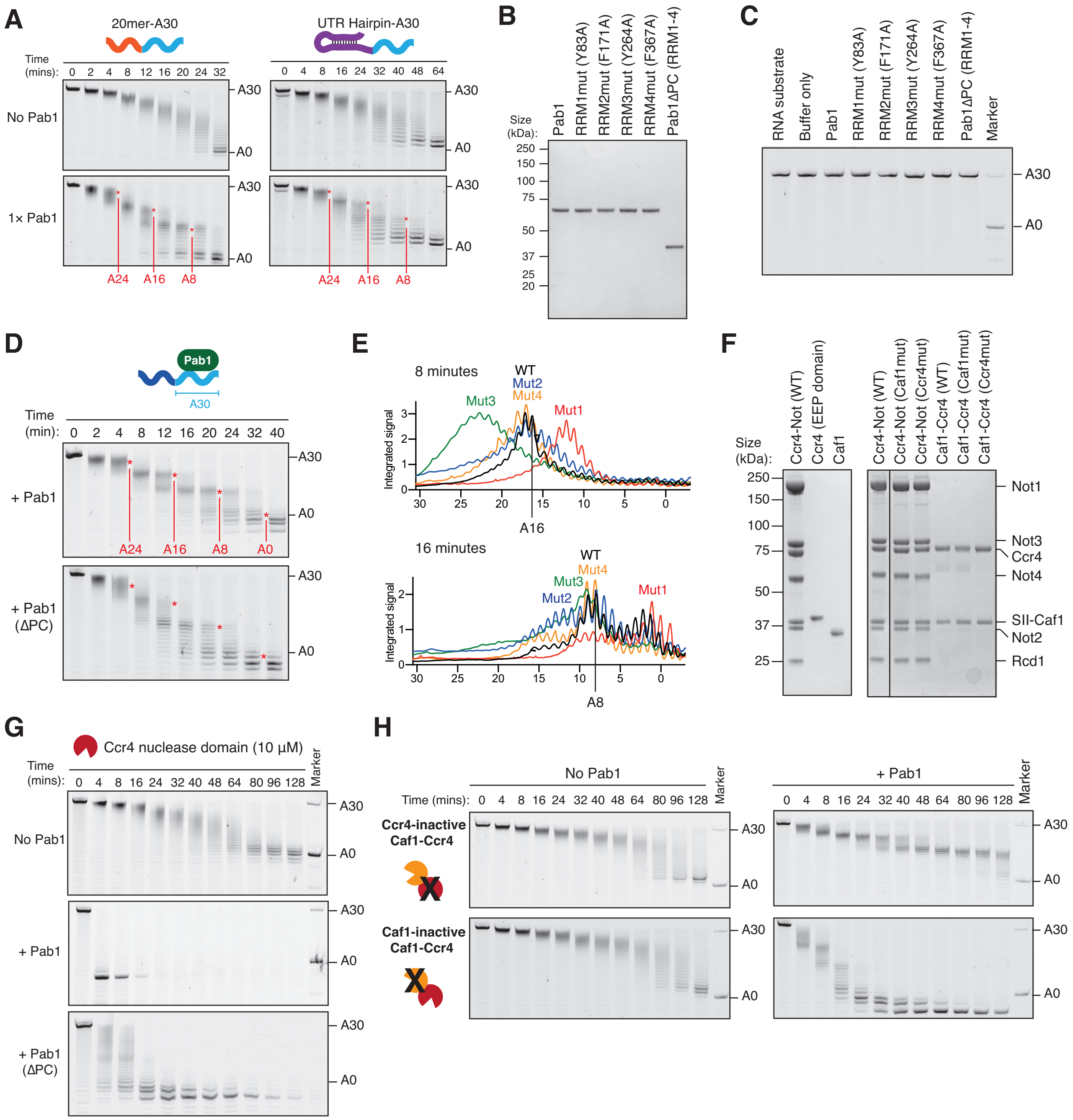

Figure S2. Pab1 differentially affects the deadenylation activities of Caf1 and Ccr4, Related to Figures 1 and 2.

(**A**) Deadenylation of a 20mer-A30 and UTR hairpin-A30 RNA by Ccr4-Not in the presence of Pab1 showing ~8 nucleotide steps. The stepwise pattern of deadenylation was also observed with these RNA substrates, indicating it is not dependent on the specific sequence of the 23mer-A30 RNA. (**B**) Coomassie-stained SDS-PAGE of purified Pab1 variant proteins. The concentration of each sample was normalized to ensure an equivalent amount of Pab1 was added to the deadenylation assays. (**C**) Control deadenylation reactions showing the absence of RNase contamination in samples of

purified Pab1 proteins. Pab1 at a concentration equivalent to that used in assays with Ccr4-Not was incubated with fluorescein-labelled 23mer-A30 RNA for 64 minutes and RNA was resolved on a denaturing polyacrylamide gel. (**D**) Deadenylation of a 30-adenosine RNA by Ccr4-Not in the presence of full-length Pab1 and a Pab1 variant containing only the RRM domains (Pab1ΔPC; residues 80–362). Asterisks indicate transient pausing of Ccr4-Not in the presence of wild-type Pab1. (**E**) Densitometric analysis of gels in Figure 1D shows altered step-wise deadenylation of 23mer-A30 RNA when mutations were introduced into the RRM domains of Pab1: RRM1 (Mut1), RRM2 (Mut2), RRM3 (Mut3), RRM4 (Mut4). Deadenylation in the presence of the RRM1 mutant is generally faster, suggesting that Pab1 is able to recruit Ccr4-Not to the RNA but RRM binding is weakened, eliminating the transient stalling of the nucleases when they encounter an RRM binding site. In contrast, mutation in RRM3 caused a slowing of deadenylation. (**F**) Coomassie-stained SDS-PAGE of purified Ccr4-Not subcomplexes and catalytic mutant variants. The concentration of enzyme in each sample was normalized to ensure an equivalent amount was added to deadenylation assays. Ccr4-Not (WT) lane reproduced from Figure S1. (**G**) Deadenylation of 23mer-A30 RNA by purified *S. pombe* Ccr4 EEP nuclease domain (10 μM) without Pab1 and in the presence of Pab1 or a truncated Pab1 variant containing only RRM domains 1-4 (Pab1ΔPC) with a 1:1 molar ratio of Pab1 to RNA. In this reaction, 10-fold more Ccr4 was added than in the reaction shown in Figure 2C, demonstrating that the enzyme is active in the absence of Pab1 but is accelerated more than 30-fold by its presence. (**H**) Deadenylation of 23mer-A30 RNA in the absence or presence of Pab1 (1:1 molar ratio to RNA) by purified *S. pombe* Caf1-Ccr4 dimeric subcomplex variants containing mutations that abolish the activity of either Caf1 or Ccr4.

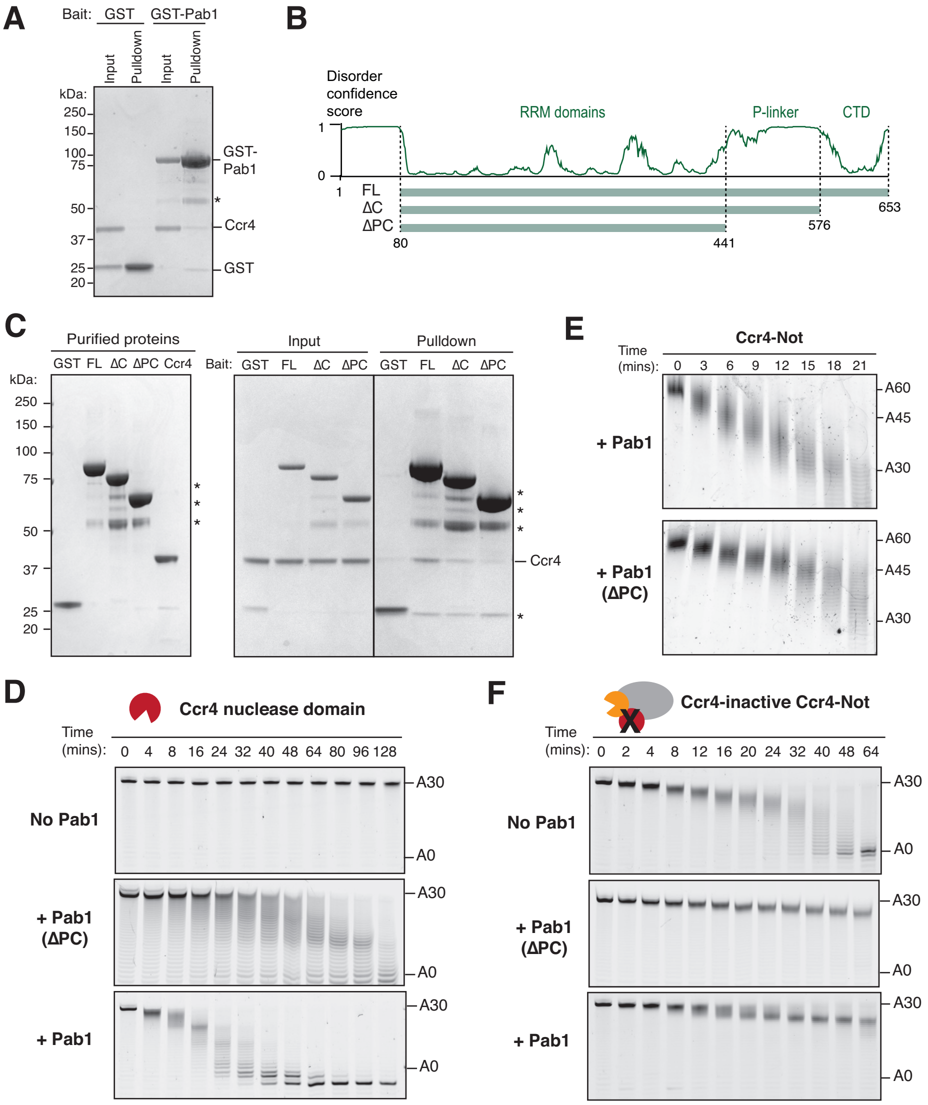

Figure S3. The C-terminal region of Pab1 interacts with Ccr4 and is important to deadenylation, Related to Figure 2.

(**A**) Pulldown assay showing the interaction between Ccr4 and immobilized GST-Pab1 is not mediated by nucleic acids. GST-Pab1 and Ccr4 were incubated with 500 U of benzonase for 30 minutes at room temperature before the experiment was performed as in Figure 2D. (**B**) Diagram showing the design of Pab1 variants based on predicted structural features of Pab1. Disorder confidence plot was generated with *DISOPRED3* (Jones and Cozzetto, 2015) and the amino acid regions of each construct are indicated. (**C**) Coomassie-stained SDS-PAGE of pull-down assays showing binding of purified Ccr4 to immobilized GST-Pab1 variants. Contaminant proteins are indicated with asterisks. (**D**) Deadenylation of 23mer-A30 RNA by Ccr4 (EEP nuclease domain; 1 μM) without Pab1, or in the presence of Pab1(ΔPC) or Pab1. Pab1-bound substrate was prepared with one Pab1 molecule per RNA. Deadenylation by isolated Ccr4 was stimulated less by a Pab1 variant lacking these domains (Pab1ΔPC) than by full-length Pab1 (Pab1: 0.94 nt/min/μmol; Pab1ΔPC: 0.23 nt/min/μmol; no Pab1: 0.04 nt/min/μmol;

also see Figure 2C). The presence of Pab1ΔPC does still increase the rate of reaction relative to when no Pab1 was added. This could be through direct interactions with Ccr4-Not and may involve allosteric effects, but a major interaction site on Pab1 is in its C-terminal region. The proline-rich linker of Pab1 had previously been shown to be important to deadenylation and mRNA stability *in vivo*. In *S. cerevisiae*, removal of this domain reduced deadenylation rates by 60–80% (Yao et al., 2007) and increased mRNA half-lives by approximately 2-fold (Simón and Séraphin, 2007). Because Pab1 self-association relies on the P-linker domain, self-association was proposed to be important to deadenylation. In our assays, the P-linker is important even in conditions when there is only one Pab1 molecule per RNA. Thus, our findings suggest that recruitment of Ccr4-Not is another critical role for the Pab1 P-linker domain. (**E**) Deadenylation of Pab1-bound A60 RNA by intact Ccr4-Not (50 nM) was impaired by removal of the C-terminal portion of Pab1 (Pab1(ΔPC)). Pab1-bound substrate was prepared with two Pab1 molecules per RNA. (**F**) Deadenylation of 23mer-A30 RNA by Ccr4-inactive Ccr4-Not (100 nM) without Pab1, or in the presence of Pab1(ΔPC) or Pab1. This shows that Pab1 RRM domains alone promote deadenylation and account for the dependence on Ccr4.

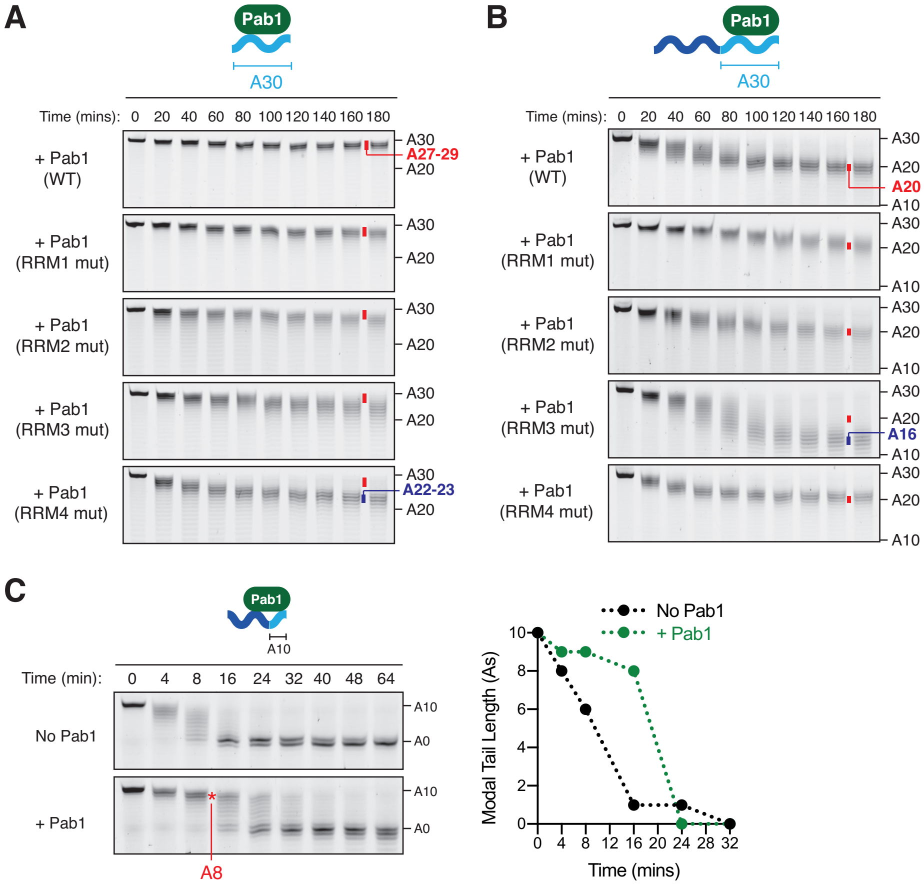

Figure S4. Mapping the position of Pab1 binding on RNA, Related to Figures 3 and 4.

(**A**) Deadenylation of a 30-adenosine RNA (without an upstream sequence) by Ccr4-inactive Ccr4-Not in the presence of Pab1 variants (1:1 molar ratio to RNA). Red markers indicate the position of the ~A28 fragment protected in the presence of wild-type Pab1. Blue marker indicates the smaller ~A22 protected fragment generated in the presence of RRM4 mutant Pab1. (**B**) Deadenylation of a 30-adenosine RNA with an upstream non-poly(A) (3′-UTR) sequence (23mer-A30) by Ccr4-inactive Ccr4-Not in the presence of Pab1 variants (1:1 molar ratio to RNA). Red markers indicate the expected position of the ~A20 protected fragment generated in the presence of wild-type Pab1. (**C**) Deadenylation of 23mer-A10 RNA by wild-type Ccr4-Not in the absence or presence of Pab1. Quantification plot of the most abundant RNA poly(A) tail length versus time is shown (right).

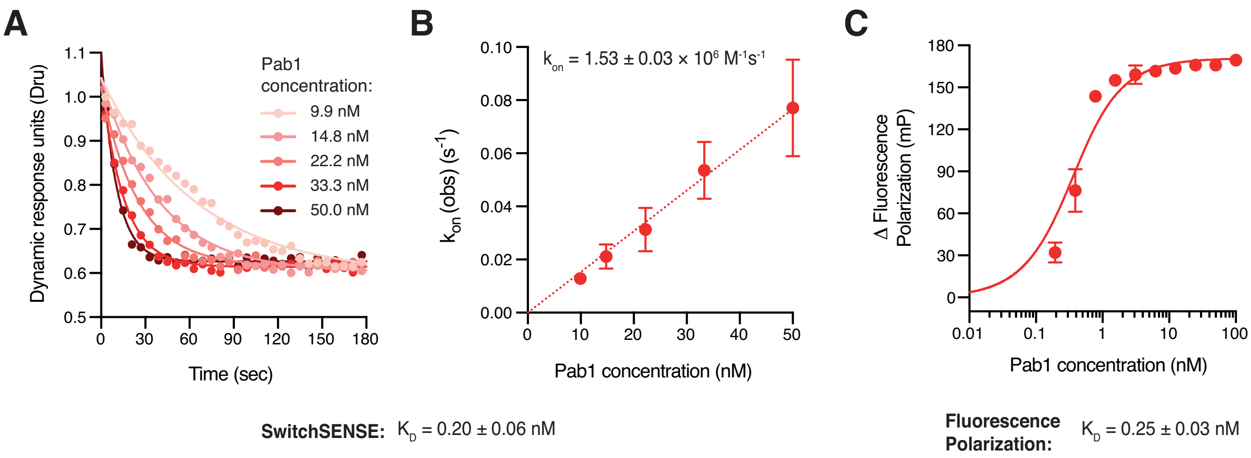

Figure S5. Kinetics of the interaction between Pab1 and polyadenosine RNA, Related to Figure 4.

(**A**) Representative switchSENSE sensograms showing the association of Pab1 with a 30-adenosine RNA at a series of protein concentrations. An exponential model was fitted to the data. (**B**) The observed association rate was determined from triplicate measurements of experiments shown in (A). Linear regression was used to determine the kinetic constant for association (k_{on}). The standard error is given. (**C**) Fluorescence polarization assay was performed with Pab1 and 5′ 6-FAM-labelled 30-adenosine RNA to validate the binding affinity (K_D) determined by switchSENSE. A one-site quadratic binding curve was fitted to the data.

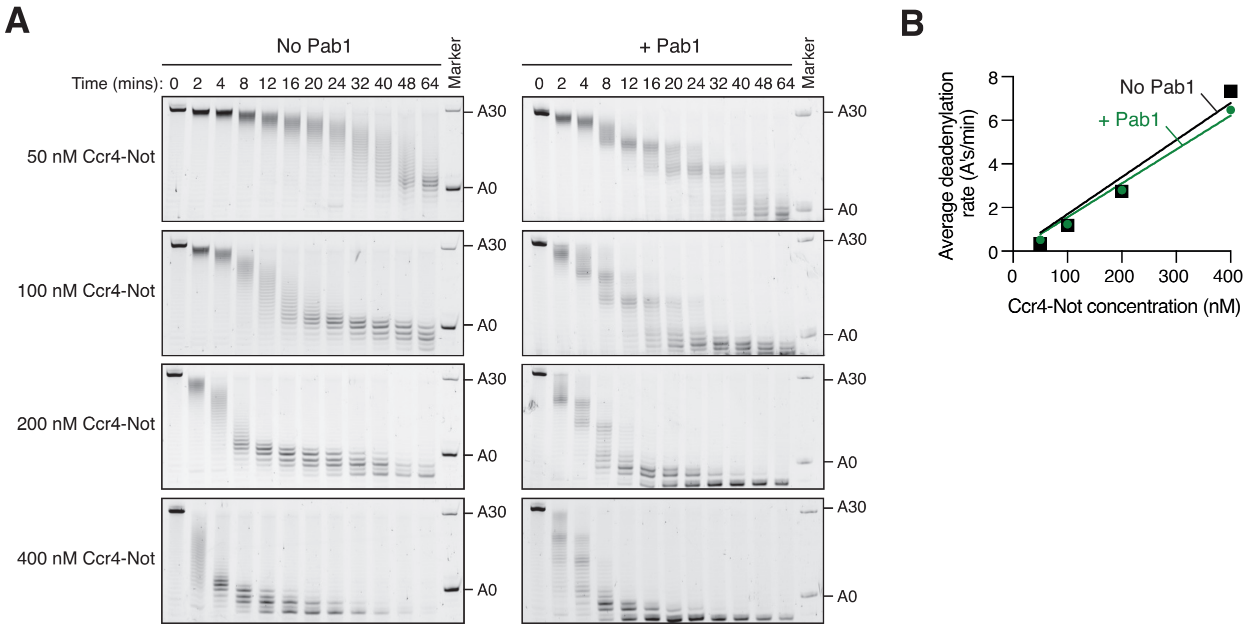

Figure S6. Pab1 does not limit the rate of Ccr4-Not-mediated deadenylation, Related to Figure 4.

(**A**) Deadenylation of 23mer-A30 RNA (200 nM) by Ccr4-Not at the indicated concentrations, in the absence or presence of Pab1 (1:1 molar ratio to RNA). (**B**) Deadenylation rates from (A) in the absence or presence of Pab1 (1:1 molar ratio to RNA) at a series of Ccr4-Not concentrations from triplicate measurements (error bars are smaller than the data points shown).

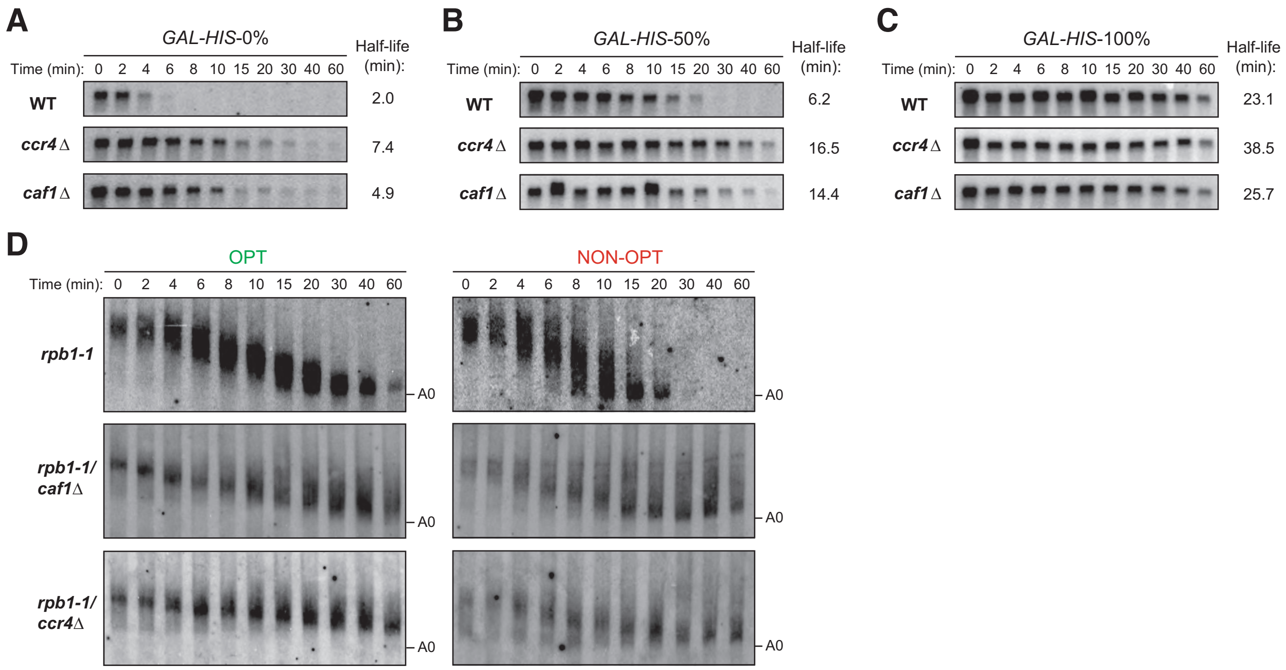

Figure S7: Caf1 preferentially destabilizes mRNAs with low codon optimality, Related to Figure 6.

(**A-C**) Northern blots of three *GAL-HIS* reporters with 0% (A), 50% (B) or 100% (C) optimality following *GAL1* transcriptional shut-off experiments in WT, *ccr4*Δ or *caf1*Δ cells. Quantification of mRNA half-life was performed following normalization to *SCR1* RNA, which is not shown. Deletion of *CAF1* preferentially stabilized mRNA with lower codon optimality (*GAL-HIS3*-0% and 50%), while deletion of *CCR4* stabilizes all three *GAL-HIS3* reporters with 0%, 50% or 100% optimality. (**D**) High-resolution polyacrylamide northern blots of the OPT and NON-OPT mRNAs following transcriptional pulse-chase experiments by inhibiting *GAL1* promoter and inactivating RNA polymerase II at 37 °C in *rpb1-1, rpb1-1/ccr4*Δ or *rpb1-1/caf1*Δ cells. A0 denotes the completely deadenylated mRNA species.

Table S1: Deadenylation rates of OPT and NON-OPT mRNAs,

Related to Figure 6.

	Deadenylation rate (nucleotides/min)*	
	OPT	NON-OPT
rpb1-1	1.70 ± 0.1	4.17 ± 0.5
*rpb1-1/caf1*Δ	1.55 ± 0.2	1.14 ± 0.1
*rpb1-1/ccr4*Δ	0.52 ± 0.1	0.88 ± 0.2

*Deadenylation rates were determined by calculating the shortest poly(A) tail length for each time point until this reaches <A10. Data are represented as the mean ± standard deviation for experiments performed in triplicate.

Table S2. RNA and DNA sequences used in this study. Related to Methods.
