# Supplementary material for: mRNA Deadenylation Is Coupled to Translation Rates by the Differential Activities of Ccr4-Not Nucleases
Source: Mol Cell. 2018 Jun 21;70(6):1089–1100.e8. doi: 10.1016/j.molcel.2018.05.033 (PMC6024076; doi:10.1016/j.molcel.2018.05.033)
Supplement: Document S2. Article plus Supplemental Information [file mmc3.pdf]

Molecular Cell

Article

mRNA Deadenylation Is Coupled to Translation Rates by the Differential Activities of Ccr4-Not Nucleases

Graphical Abstract

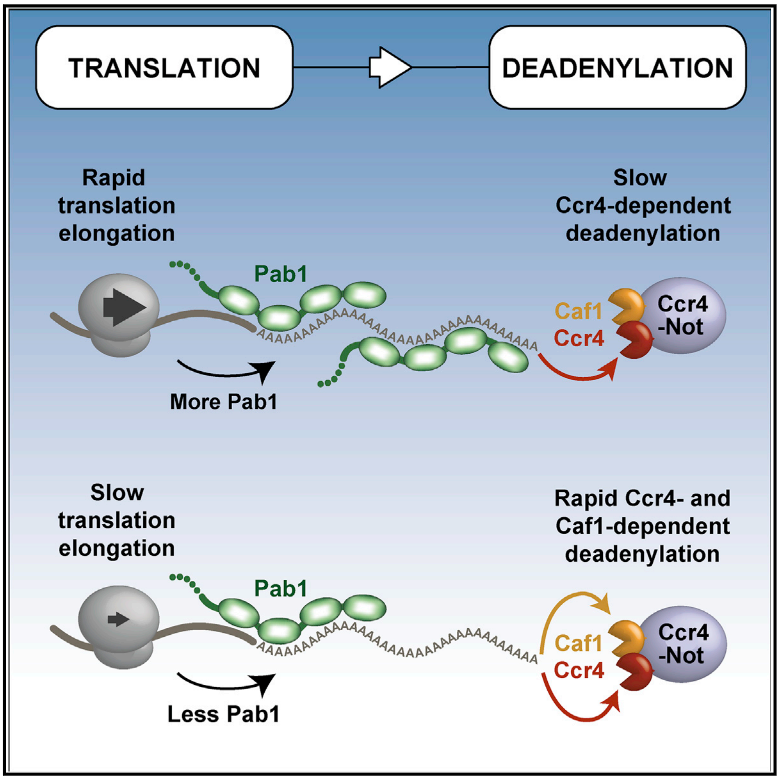

Authors

Michael W. Webster, Ying-Hsin Chen, James A.W. Stowell, ..., Brenton R. Graveley, Jeff Coller, Lori A. Passmore

Correspondence

jmc71@case.edu (J.C.),
passmore@mrc-lmb.cam.ac.uk (L.A.P.)

In Brief

Ccr4-Not is a conserved deadenylation complex that contains two exonucleases, Ccr4 and Caf1. Webster et al. show that Ccr4 is a general deadenylase that removes Pab1-bound poly(A) tails. In contrast, Caf1 is a specialized exonuclease that acts on transcripts with low codon optimality, coupling translation elongation and mRNA stability.

Highlights

- Poly(A)-binding protein is efficiently released by Ccr4-Not nuclease activity
- Ccr4, but not Caf1, removes poly(A) tails bound to Pab1
- Ccr4 acts on all transcripts and Caf1 acts on transcripts with low codon optimality
- Deadenylation by Ccr4-Not connects translation with mRNA stability

Webster et al., 2018, Molecular Cell *70*, 1089–1100
June 21, 2018 © 2018 MRC Laboratory of Molecular Biology.
Published by Elsevier Inc.
https://doi.org/10.1016/j.molcel.2018.05.033

mRNA Deadenylation Is Coupled to Translation Rates by the Differential Activities of Ccr4-Not Nucleases

Michael W. Webster,[1] Ying-Hsin Chen,[2] James A.W. Stowell,[1] Najwa Alhusaini,[2] Thomas Sweet,[2] Brenton R. Graveley,[3] Jeff Coller,[2,*] and Lori A. Passmore[1,4,*]
[1]MRC Laboratory of Molecular Biology, Cambridge CB2 0QH, UK
[2]The Center for RNA Science and Therapeutics, Case Western Reserve University, Cleveland, OH 44106-4960, USA
[3]Department of Genetics and Developmental Biology, Institute for Systems Genomics, University of Connecticut Health Center, Farmington, CT 06030, USA
[4]Lead Contact
*Correspondence: jmc71@case.edu (J.C.), passmore@mrc-lmb.cam.ac.uk (L.A.P.)
https://doi.org/10.1016/j.molcel.2018.05.033

SUMMARY

Translation and decay of eukaryotic mRNAs is controlled by shortening of the poly(A) tail and release of the poly(A)-binding protein Pab1/PABP. The Ccr4-Not complex contains two exonucleases—Ccr4 and Caf1/Pop2—that mediate mRNA deadenylation. Here, using a fully reconstituted biochemical system with proteins from the fission yeast *Schizosaccharomyces pombe*, we show that Pab1 interacts with Ccr4-Not, stimulates deadenylation, and differentiates the roles of the nuclease enzymes. Surprisingly, Pab1 release relies on Ccr4 activity. In agreement with this, *in vivo* experiments in budding yeast show that Ccr4 is a general deadenylase that acts on all mRNAs. In contrast, Caf1 only trims poly(A) not bound by Pab1. As a consequence, Caf1 is a specialized deadenylase required for the selective deadenylation of transcripts with lower rates of translation elongation and reduced Pab1 occupancy. These findings reveal a coupling between the rates of translation and deadenylation that is dependent on Pab1 and Ccr4-Not.

INTRODUCTION

The 3′ poly(A) tail of eukaryotic mRNAs is a central determinant of gene expression. The conserved cytoplasmic poly(A)-binding protein (Pab1 in yeast/PABPC1 in mammals) binds to the poly(A) tail with high affinity. Pab1/PABPC1 interacts with translation initiation factors to promote mRNA translation and also protects mRNAs from degradation (Caponigro and Parker, 1995; Coller et al., 1998; Sachs and Davis, 1989). Consistent with this, shortening of the poly(A) tail (deadenylation) and release of Pab1 repress gene expression by reducing translation and mRNA stability. Deadenylation can be stimulated by miRNAs, mRNA-binding proteins, and covalent RNA modifications. Despite the central role of deadenylation in gene expression, it is unclear how exonucleases gain access to a poly(A) tail that is concealed by tightly bound Pab1.

Deadenylation is catalyzed by Ccr4-Not and Pan2-Pan3 (Tucker et al., 2001; Wahle and Winkler, 2013). Ccr4-Not, which is thought to play the major role, contains seven core subunits, including two poly(A)-selective exonucleases, Ccr4 and Caf1/Pop2 (Parker, 2012). Ccr4-Not is recruited to specific mRNAs to direct rapid deadenylation during diverse biological processes, including embryogenesis, immunological responses, and cell proliferation (Belloc and Méndez, 2008; Carballo et al., 1998; Subtelny et al., 2014). Targeted deadenylation occurs when proteins bound to specific mRNA sequences recruit the Ccr4-Not complex (Goldstrohm et al., 2006; Stowell et al., 2016; Wahle and Winkler, 2013). The action of these mRNA-binding proteins does not, however, fully account for the wide range of half-lives observed across the eukaryotic transcriptome (Cheng et al., 2017). An additional major determinant of mRNA decay is the rate of translation elongation, and this correlates with codon optimality (Presnyak et al., 2015). The coupling between translation and mRNA stability depends on the DEAD-box helicase Dhh1/DDX6 (Radhakrishnan et al., 2016) and is linked to deadenylation (Bazzini et al., 2016; Mishima and Tomari, 2016; Presnyak et al., 2015). Yet, the molecular mechanisms whereby the RNA decay machinery senses translation rates remain largely enigmatic.

The two nucleases of Ccr4-Not have similar enzymatic activities *in vitro* and overlapping roles in deadenylation *in vivo*. Purified human, mouse, and yeast Ccr4 and Caf1 are poly(A)-specific nucleases (Bianchin et al., 2005; Daugeron et al., 2001; Thore et al., 2003; Tucker et al., 2002; Viswanathan et al., 2004; Wang et al., 2010). We recently demonstrated that both nucleases are active when they are integrated into an intact recombinant *Schizosaccharomyces pombe* (*S. pombe*) Ccr4-Not complex, and active site mutations in either nuclease have only minor effects on the overall activity (Stowell et al., 2016). Deletion of Ccr4 in *Saccharomyces cerevisiae* (*S. cerevisiae*) does, however, impair rates of poly(A) tail shortening more than deletion of Caf1 (Tucker et al., 2001). Furthermore, the nucleases are structurally dissimilar: Ccr4 is a member of the

This is an open access article under the CC BY license (http://creativecommons.org/licenses/by/4.0/).

endonuclease-exonuclease-phosphatase (EEP) family, while Caf1 adopts an RNase D fold. It is therefore unclear whether the nucleases of Ccr4-Not have separable enzymatic functions and whether they are differentially regulated by additional factors.

Pab1/PABPC1 contains four RNA recognition motif (RRM) domains that together bind poly(A) RNA with low nanomolar affinity (Kühn and Pieler, 1996). A C-terminal region, comprised of a proline-rich linker (P-linker) and C-terminal domain (CTD), mediates self-association and interactions with other proteins (Mangus et al., 2003). RRMs 1 and 2 are thought to bind with higher affinity and be more specific for poly(A) than RRMs 3 and 4 (Burd et al., 1991; Kühn and Pieler, 1996).

A current model suggests that a conserved role of Pab1/PABPC1 is to conceal the 3′ end of mRNAs, protecting them from Ccr4-Not (Lee et al., 2010; Parker, 2012; Yamashita et al., 2005). Consistent with this, excess Pab1 was reported to inhibit the activity of purified *S. cerevisiae* Ccr4 and Caf1 using *in vitro* assays (Simón and Séraphin, 2007; Tucker et al., 2002; Viswanathan et al., 2004). It is therefore possible that other proteins release Pab1 to permit deadenylation (Khaleghpour et al., 2001; Weidmann et al., 2014; Zekri et al., 2013). Recent data showing that PABPC1 occupancy on mRNAs is not correlated with steady-state poly(A) tail lengths may support this model (Rissland et al., 2017; Zekri et al., 2013). Contrary to the view of Pab1 as an inhibitor of deadenylation, Pab1-deficient yeast have a reduced rate of poly(A) shortening (Caponigro and Parker, 1995). Furthermore, PABPC1 was required for efficient miRNA-induced deadenylation in a mouse cell extract system (Fabian et al., 2009). Since Pab1/PABPC1 is an essential gene with pleiotropic functions, studying its role in deadenylation *in vivo* is a particular challenge.

Here, we use a fully reconstituted biochemical system to investigate the activity of *S. pombe* Ccr4-Not on Pab1-bound RNA and elucidate how Pab1 is released. Surprisingly, Pab1 differentiates the activities of the two Ccr4-Not nucleases: only Ccr4 is capable of shortening poly(A) tails bound by Pab1. *In vivo*, we find that *S. cerevisiae* transcripts with optimal codons have higher Pab1 occupancy, undergo slow poly(A) tail removal, and are not dependent on Caf1 for deadenylation. Collectively, our findings reveal a functional distinction between the deadenylase enzymes of Ccr4-Not and provide mechanistic insight into the coupling between translation, mRNP (messenger ribonucleoprotein) complex composition, and mRNA stability.

RESULTS

Pab1 Accelerates Shortening of the Poly(A) Tail by Ccr4-Not

To study the effect of Pab1 on Ccr4-Not activity and to determine how Pab1 is released from mRNAs, we reconstituted this process *in vitro*. Recombinant *S. pombe* Ccr4-Not complex was isolated from insect cells overexpressing all seven core subunits (Stowell et al., 2016) (Figure S1A). To approximate physiological conditions, we used a model RNA substrate with a short upstream "3′ UTR" sequence followed by 60 adenosines (20-mer-A60) (Webster et al., 2017). We loaded purified Pab1 onto this RNA with controlled stoichiometry (two Pab1 molecules/RNA) and verified this using electrophoretic mobility shift assays (Figures S1B and S1C). When purified Ccr4-Not was incubated with the model RNA substrate, the poly(A) tail was removed (Figure 1A). Surprisingly, the average deadenylation rate was increased 3-fold when Pab1 was present (Figure S1D). Thus, the activity of Ccr4-Not is not restricted by Pab1 binding tightly to the poly(A) RNA substrate.

One mechanism whereby Pab1 could increase the rate of deadenylation is by direct interaction with the Ccr4-Not complex, recruiting it to the RNA substrate. To test this, we used pull-down assays and found that purified Ccr4-Not bound to immobilized GST-Pab1 (Figure 1B). Thus, the rate of deadenylation by Ccr4-Not is likely accelerated by Pab1 due to a direct physical interaction between these factors.

Previous reports that Pab1 inhibits deadenylation by isolated Ccr4 (Tucker et al., 2001) led to the prevailing view that Pab1 protects against the removal of the poly(A) tail. In those experiments, however, Pab1 was present in a 4- to 400-fold molar excess over RNA. Consistent with this, we found that addition of excess Pab1 inhibited deadenylation by Ccr4-Not approximately 2-fold (Figure S1E). Excess Pab1 did not inhibit deadenylation of a substrate lacking an upstream sequence (Figure S1E). Thus, excess Pab1 likely bound to the non-poly(A) sequence upstream of the poly(A) tail (Figure S1C). Together, these data show that excess Pab1 can slow deadenylation when the RNA substrate contains a 3′ UTR, and it does not inhibit deadenylation by concealing the poly(A) tail. Instead, when it is stoichiometrically loaded onto poly(A) tails, Pab1 can promote removal of A60 tails, likely through a direct interaction with Ccr4-Not.

Deadenylation Reveals the 3′ Ends of Pab1 RRM Footprints

Deadenylation in the presence of Pab1 was not uniform along the length of the poly(A) tail and appeared to proceed in a series of steps (Figure 1A). To analyze this at higher, single-nucleotide resolution, we used an RNA substrate with a 30-adenosine tail that bound one Pab1 molecule. Pab1 did not largely affect the average rate at which the 30-adenosine tail was shortened, but it caused the accumulation of three different RNA species, separated in size by ~8 nucleotides (A8, A16, and A24) (Figures 1C, S1D, and S2A). Hence, Pab1 causes deadenylation to proceed stepwise, pausing at defined and regularly spaced intervals.

We hypothesized that the steps in deadenylation are caused by protection of the poly(A) tail by the RRM domains of Pab1. The position of the steps would therefore represent the 3′ end of an RRM domain footprint on RNA, defined by the binding site of the RRM and steric constraints imposed by the colliding nuclease and Pab1. Consistent with this, deadenylation also proceeded in ~8 nucleotide steps in the presence of a truncated Pab1 protein containing only the four RRMs (Figures S2B–S2D).

To understand the contribution of each RRM, we generated four Pab1 variants, each with a mutation that impairs the binding of RNA to one RRM domain (Deardorff and Sachs, 1997). A mutation that impairs RNA binding to RRM 4 had no effect on the deadenylation activity of Ccr4-Not (Figure 1D). Mutation in RRM 2 had a moderate effect, reducing the prominence of the

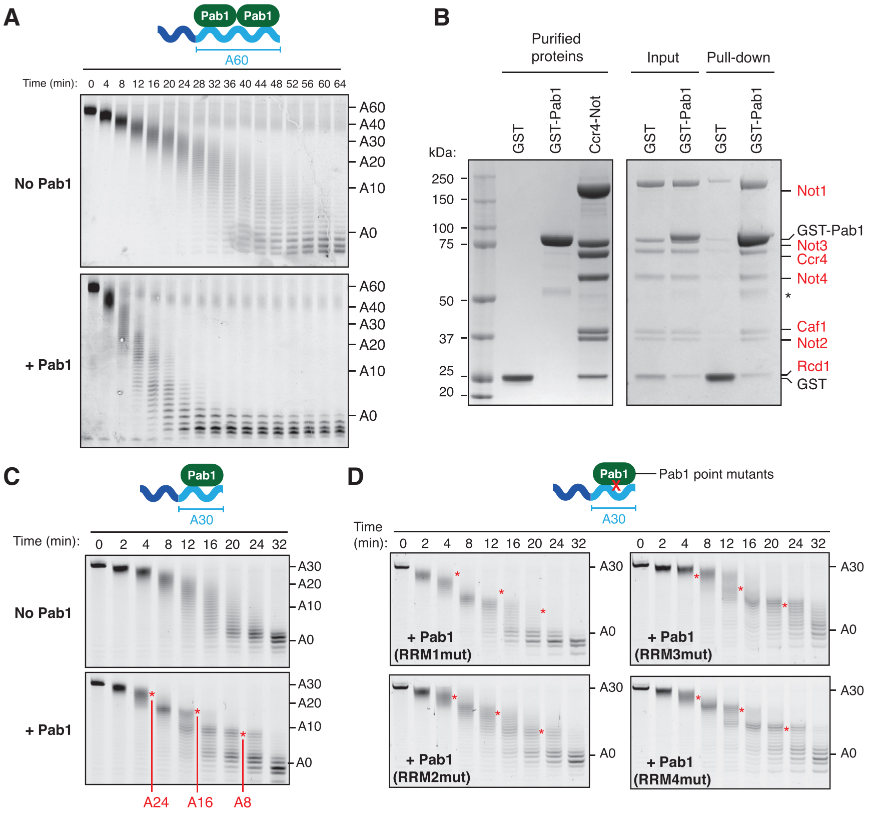

Figure 1. Pab1 Stimulates Stepwise Deadenylation by Ccr4-Not

(A) Deadenylation by purified Ccr4-Not in the presence and absence of Pab1. The RNA substrate comprises 20 non-poly(A) nucleotides followed by a 60-adenosine poly(A) tail. RNA products (4-min time points) were resolved on a denaturing polyacrylamide gel. Pab1-bound substrates were prepared with two Pab1 molecules per RNA.

(B) Coomassie-stained SDS-PAGE of pull-down assay showing binding of purified Ccr4-Not (red labels) to immobilized GST-Pab1. Purified proteins (before mixing), Input (proteins mixed before loading on resin), and Pull-down (proteins bound to resin after washing) are shown. The asterisk indicates a contaminant protein.

(C) Deadenylation of 5′ fluorescently labeled 23-mer-A30 RNA substrate. Pab1-bound substrates were prepared with one Pab1 molecule per RNA. Poly(A) tail lengths are indicated, and RRM footprints are marked with red asterisks.

(D) Deadenylation of 5′ fluorescently labeled 23-mer-A30 RNA substrates in the presence of Pab1 variants. The positions of footprints observed with wild-type Pab1 in (C) are indicated with red asterisks.

See also Figures S1 and S2.

footprints. In contrast, mutation of RRM 1 or 3 produced more substantial changes in the pattern of footprints, reducing the stepwise nature of deadenylation (Figure S2E). It is likely that mutation of one RRM influences RNA binding by adjacent RRMs, thereby leading to these complex effects on deadenylation. Based on these results, we propose that in wild-type Pab1, RRMs 1, 2, and 3 each protect ~8 nucleotides of the poly(A) tail, and Ccr4-Not stalls when it encounters each one.

Previous studies had shown that RRM 4 is not selective for poly(A) (Burd et al., 1991). Given that we do not observe a footprint for RRM 4 in deadenylation reactions (Figure 1D), this domain may be bound to the non-poly(A) 3′ UTR of the RNA rather than the poly(A) tail. The Pab1 molecule at the 5′ end of the poly(A) tail may therefore bind across the junction of the 3′ UTR and poly(A). This model is consistent with transcriptome-wide mapping of Pab1 binding sites in yeast, which showed that Pab1 binds non-poly(A) sequences and that RRM 4 is the primary site of protein-RNA crosslinks (Baejen et al., 2014; Kramer et al., 2014; Tuck and Tollervey, 2013). This also suggests the possibility that the 3′ UTR sequence could further influence Pab1 positioning.

Shortening of the Pab1-Bound Poly(A) Tail Is Catalyzed by Ccr4, but Not Caf1

We recently showed that both nucleases in Ccr4-Not mediate *in vitro* deadenylation of a model RNA (Stowell et al., 2016). To investigate whether the activities of Ccr4 and Caf1 are similarly redundant on Pab1-bound RNAs, we used purified Ccr4-Not variants with point mutations that abolish the catalytic activity of either nuclease (Figure S2F). Caf1 inactivation did not change *in vitro* deadenylation by Ccr4-Not (Figure 2A). In contrast, in the presence of Pab1, a complex containing inactive Ccr4 did not fully deadenylate substrate RNAs with a 30-adenosine tail (Figure 2A). A product with ~22 adenosines accumulated but only in the presence of Pab1. Thus, surprisingly, removal of the Pab1-bound poly(A) tail required the catalytic activity of Ccr4, but not that of Caf1. The 22 nucleotides resistant to deadenylation likely represent the footprint of Pab1 on poly(A) RNA.

To test whether Ccr4 or Caf1 is required for removal of the Pab1-bound poly(A) tail *in vivo*, we examined global poly(A) tail shortening following transcriptional shut-off in *S. cerevisiae* strains containing a deletion of either *CCR4* or *CAF1*. Whereas wild-type yeast showed a continuous range of poly(A) tail lengths that shorten with time, the *ccr4*Δ strain accumulated poly(A) tails of ~30 adenosines and had a marked reduction of poly(A) tails shorter than ~22 adenosines (Figure 2B). Based on the similarity in the size of this protected region to that observed with the Ccr4 mutant complex *in vitro*, this likely represents a footprint of Pab1 *in vivo*. Deletion of *CAF1* did not generate a similar footprint (Figure 2B).

Pab1 Stimulates Isolated Ccr4 but Inhibits Isolated Caf1

We tested whether the requirement for Ccr4 to deadenylate Pab1-bound poly(A) tails is a property of the intact Ccr4-Not complex or intrinsic to the nuclease subunits. Purified, isolated

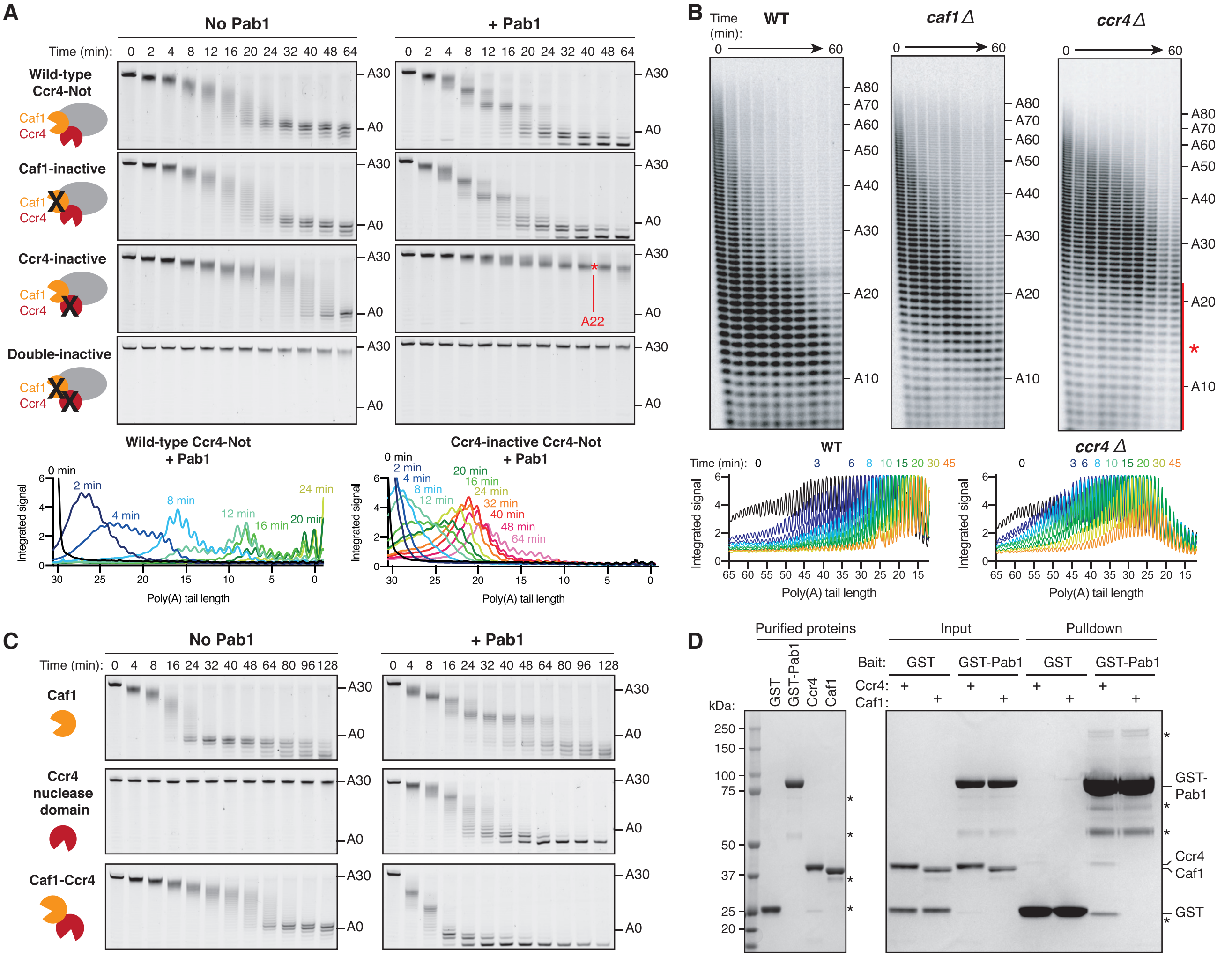

Figure 2. Shortening of Pab1-Bound Poly(A) Tails Is Catalyzed by Ccr4

(A) Deadenylation of a 23-mer-A30 RNA in the absence or presence of Pab1 by Ccr4-Not and variant complexes with mutations in the active site of Ccr4 (Ccr4-inactive), Caf1 (Caf1-inactive), or both Ccr4 and Caf1 (double-inactive). Densitometric analyses were performed on selected gels (bottom).

(B) Global poly(A) tail length in wild-type (WT) *S. cerevisiae* and strains containing deletion of *CCR4* or *CAF1*. The red asterisk indicates incomplete deadenylation in the *ccr4*Δ strain. Densitometric analyses were performed on selected gels (bottom).

(C) Deadenylation of a 23-mer-A30 RNA by isolated Caf1 protein, Ccr4 (EEP nuclease domain), or the Caf1-Ccr4 heterodimer.

(D) Coomassie-stained SDS-PAGE of pull-down assays showing binding of purified Ccr4 or Caf1 to immobilized GST-Pab1. Contaminant proteins are indicated with asterisks.

In (A) and (C), Pab1-bound substrate was prepared with one Pab1 molecule per RNA. See also Figures S2 and S3.

nucleases were >10-fold less active than the Ccr4-Not complex, necessitating the use of higher protein concentrations in the assays. Pab1 inhibited the activity of purified Caf1, reducing the average rate of deadenylation by 3-fold (Figure 2C). Importantly, however, deadenylation by Caf1 was not halted at ~22 adenosines like Ccr4-inactive Ccr4-Not. This indicates that additional subunits of the Ccr4-Not complex regulate the activity of Caf1 and enforce the dependence on Ccr4 for the removal of adenosines bound by Pab1.

In contrast, the activity of the isolated nuclease domain of Ccr4 was strongly stimulated by the presence of Pab1 (Figures 2C and S2G). Pab1 also stimulated a heterodimeric complex consisting of only Caf1 and Ccr4, and Ccr4 activity was required for this effect (Figures 2C and S2H). Hence, the overall effect of Pab1 on each deadenylase enzyme is similar whether they are isolated or integrated into the intact Ccr4-Not complex. However, the activity of Caf1 is more restricted when it is part of Ccr4-Not, indicating that non-enzymatic subunits of the complex play a regulatory role.

Ccr4 Interacts with Pab1

Since Pab1 influences the activities of the isolated nucleases of Ccr4-Not, we tested whether Pab1 interacts directly with Ccr4 and Caf1. In pull-down assays, GST-Pab1 interacted weakly

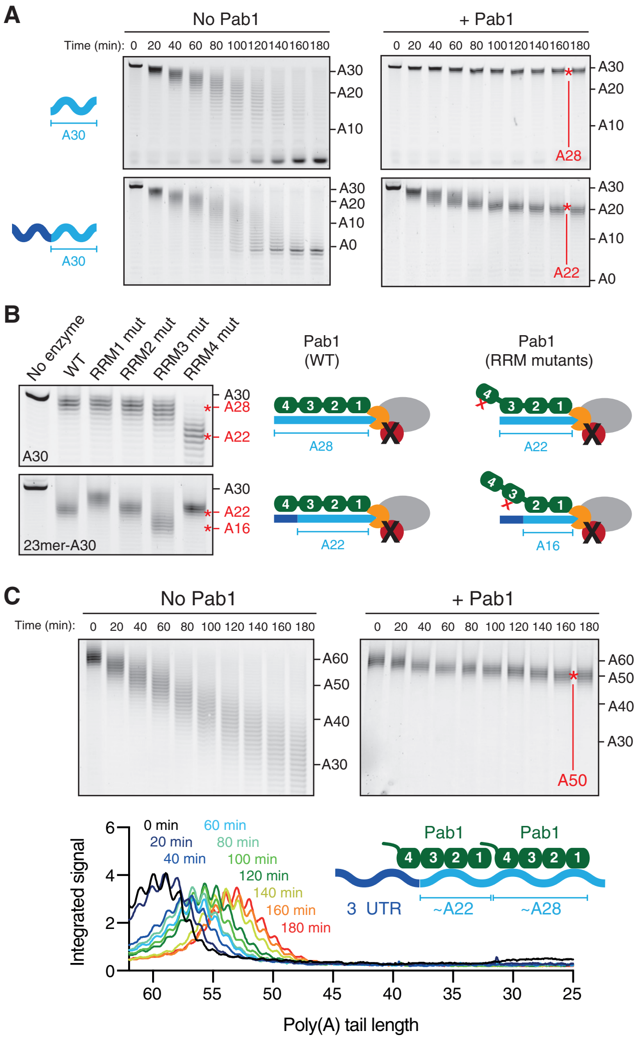

Figure 3. Pab1 Organization on the Poly(A) Tail

(A) Deadenylation by Ccr4-inactive Ccr4-Not to map Pab1-binding site on A30 and 23-mer-A30 RNA substrates. Red asterisks indicate accumulated product poly(A) tail lengths.

(B) Deadenylation reaction end points (180 min) following addition of Ccr4-inactive Ccr4-Not to A30 (top) and 23-mer-A30 (bottom) RNA substrates in the presence of the indicated Pab1 variants. Red asterisks indicate accumulated product poly(A) tail lengths. Full time courses are shown in Figures S4A and S4B. Models of Pab1 binding to each RNA are shown on the right.

(C) Deadenylation by Ccr4-inactive Ccr4-Not on 20-mer-A60 RNA in the absence or presence of Pab1 (2:1 molar ratio to RNA). Densitometric analysis of the reaction with Pab1 shows that the protected RNA fragment is ~50–55 adenosines. A model for Pab1-RNA binding is shown.

See also Figure S4.

but reproducibly with the nuclease domain of Ccr4, but not with Caf1 (Figures 2D and S3A). This likely accounts for the stimulation of isolated Ccr4 activity by Pab1.

The C-terminal portion of Pab1 comprising the proline-rich linker and the CTD was important for interaction with Ccr4 and its stimulatory effect on deadenylation (Figures S3B–S3E). Still, Ccr4 activity was required for Ccr4-Not-mediated release of Pab1 lacking the P-linker and CTD (Figure S3F).

Ccr4-Inactive Ccr4-Not Reveals the Organization of Pab1 on the Poly(A) Tail

Since the Caf1 exonuclease in Ccr4-inactive complex stops when it encounters nucleotides bound by Pab1, we could use this complex to map the Pab1 binding site on RNAs. An RNA containing 30 adenosines (lacking the upstream 3′ UTR sequence) was not efficiently deadenylated by Ccr4-inactive complex (Figure 3A). This is consistent with the ~27-nucleotide footprint of Pab1 observed when cellular mRNA poly(A) tails were digested with RNase T2 (Baer and Kornberg, 1983). In contrast, the footprint of Pab1 on the poly(A) tail of an RNA with an upstream 3′ UTR is ~22 nucleotides (Figure 3A). Hence, the 3′ UTR alters the position of Pab1 binding to an A30 sequence, allowing more adenosines to be enzymatically removed by Caf1. This finding supports our model in which Pab1 RRMs 1–3 bind selectively to poly(A), while RRM 4 instead binds to the 3′ UTR. Pab1 molecules that do not have access to a 3′ UTR bind poly(A) with all four RRMs and consequently generate a longer poly(A) footprint.

Experiments with Pab1 variants containing mutations in each RRM domain provided further support for this model: RRM 4 mutation resulted in a reduction of the size of the protected sequence from 28 to 22 nucleotides on the A30 RNA but had no effect on a substrate with an upstream 3′ UTR sequence (Figures 3B and S4A). The size of the footprint on the substrate with an upstream 3′ UTR was instead reduced from 22 to 16 adenosines by a mutation in RRM 3 (Figures 3B and S4B).

Two Pab1 molecules on a 60-adenosine substrate protect 50–55 adenosines (Figure 3C). This corresponds to the sequence protected by RRMs 1–3 of a Pab1 molecule that is bound proximal to the 3′ UTR (~22 nucleotides) plus the sequence that is protected if all four RRMs bind poly(A) (~28 nucleotides; Figure 3C). We note that the arrangement of Pab1 on RNA, with RRM 4 bound to the 3′ UTR, may not exist prior to the onset of deadenylation: both molecules of Pab1 could initially bind poly(A) with all four RRM domains, but as the tail is shortened, the UTR-proximal molecule is forced into the non-poly(A) region.

The 3′ UTR Can Stabilize Pab1 Binding

Pab1 release from RNA is important for control of translation and RNA stability *in vivo*. Because Ccr4-Not deadenylates Pab1-bound poly(A) tails, it can release Pab1, yet it is not known how many nucleotides must be removed before this occurs. It was previously reported that the shortest poly(A) sequence that Pab1 binds with high affinity is ~12 nucleotides (Kühn and Pieler, 1996; Sachs et al., 1987). In our experiments, the accumulation of RNA products with tail lengths of ~8 adenosines indicated that Pab1 influences deadenylation even when the poly(A) tail is shortened to this length (Figure 1). Consistent with this, Pab1 generated a prominent 8-nt deadenylation footprint when a model mRNA with a short, 10-adenosine tail was used as a substrate (Figure S4C).

To investigate whether Pab1 binding to the poly(A) tail can be stabilized through interactions with upstream 3′ UTR sequences, we compared the affinities of Pab1 for A12 RNAs with and without a 10-nt non-poly(A) upstream sequence. Using fluorescence polarization assays, we found that Pab1

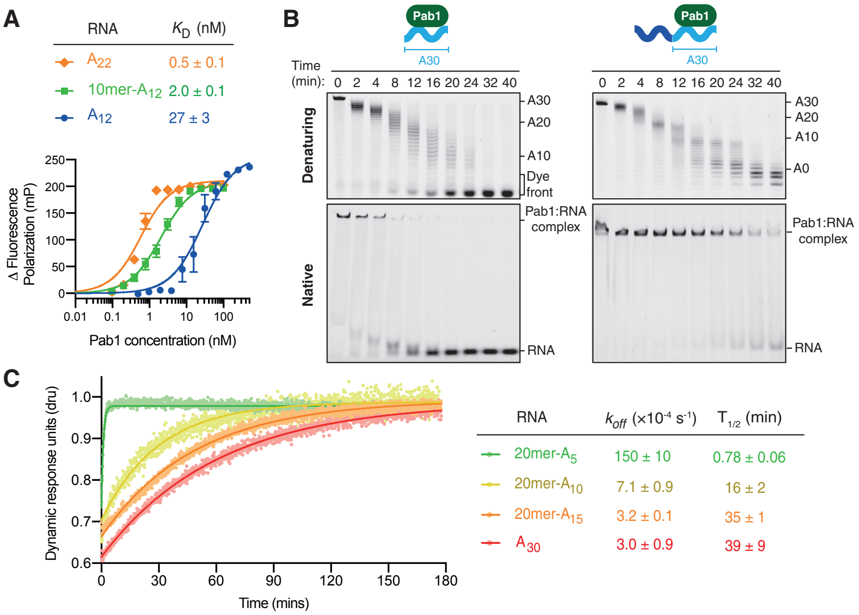

Figure 4. Ccr4-Not Releases Pab1 from Short Poly(A) Tails

(A) Fluorescence polarization assay showing interaction of Pab1 with 5′ 6-FAM-labeled A22, 10-mer-A12, and A12 RNAs. Error bars are standard error (n = 3 for A12; n = 5 for A22 and 10-mer-A12). K_Ds are represented as the mean ± standard error.

(B) Deadenylation of A30 and 23-mer-A30 RNAs by Ccr4-Not analyzed by both denaturing PAGE (upper gels) and native PAGE (lower gels). Samples were collected from the same reaction at the indicated time points after addition of Ccr4-Not to allow a direct comparison between RNA product sizes and Pab1 binding, respectively. Pab1-bound substrate was prepared with one Pab1 molecule per RNA. Upper right panel is reproduced from Figure 1C for comparison.

(C) Representative SwitchSENSE sensograms showing the dissociation of Pab1 from the indicated RNA sequences. Rate constants and half-lives for dissociation with standard error are shown for measurements performed in triplicate.

See also Figures S4–S6.

binds the 10-mer-A12 RNA with substantially higher affinity than A12 alone (K_D ~2.0 nM and ~27 nM, respectively; Figure 4A). Pab1 binds an A22 RNA with even higher affinity (K_D ~0.5 nM). Hence, the interaction of Pab1 with short poly(A) RNA is stabilized by an upstream non-poly(A) sequence.

Ccr4-Not Does Not Release Pab1 until the Poly(A) Tail Is <10 Nucleotides

To measure the dissociation of Pab1 during poly(A) removal, we stopped the magnesium-dependent deadenylation reaction at a series of time points with EDTA and separated Pab1-bound RNA from unbound RNA by native gel electrophoresis. Pab1 dissociated from RNA without an upstream 3′ UTR sequence when the tail was shortened to ~20 adenosines (Figure 4B, left). In contrast, Pab1 remained stably associated with the UTR-containing RNA until the final ~8 adenosines were removed (Figure 4B, right). The 3′ UTR sequence therefore affects deadenylation by anchoring Pab1 to the proximal region of the poly(A) tail, stabilizing its binding even when the poly(A) tail is less than 10 adenosines.

We sought to understand the kinetics of Pab1 dissociation using SwitchSENSE (Figure 4C). In this technique, fluorescently labeled DNA nanolevers hybridized to an RNA of interest are oscillated by an alternating electric field. The speed at which the nanolevers respond to the changing electric potential is reduced upon binding of protein (Cléry et al., 2017). By monitoring this in real time, we measured the dissociation rates of Pab1 from RNAs with a 3′ UTR and poly(A) tails of 5, 10, or 15 adenosines. The k_{off} was over an order of magnitude faster on an RNA substrate with a 5-adenosine tail compared to a 10-adenosine tail. This agrees with Pab1 binding to RNA during deadenylation assays (Figure 4B) and is consistent with Pab1 dissociating from mRNAs when the poly(A) tail is shortened to 5–10 adenosines. As a single RRM footprints ~8 adenosines, it is likely that Pab1 release generally occurs once the poly(A) tail has been shortened beyond this length.

The overall binding affinity (K_D) of Pab1 for 30-adenosine RNA was ~0.2 nM (Figure S5). The half-life for dissociation is approximately 40 min (Figure 4C). In contrast, the Pab1-bound poly(A) tail can be removed in under 5 min in conditions of excess Ccr4-Not (Figure S6). Thus, dissociation of Pab1 from the poly(A) tail does not appear to be inherently rate limiting to deadenylation *in vitro*. Instead, the exonuclease activity of Ccr4-Not releases Pab1 by shortening the poly(A) sequence to which Pab1 binds.

Codon Optimality Is Correlated with Pab1 Association with mRNA

Pab1 is thought to be associated with the mRNAs of all genes, but recent data suggest that Pab1 occupancy varies in a gene-dependent manner (Costello et al., 2015). For example, Pab1 is enriched on mRNAs encoding ribosomal proteins and on transcripts with high ribosome occupancy. This is particularly striking because short poly(A) tails are a general feature of highly expressed transcripts (Lima et al., 2017). Since highly expressed transcripts often have more optimal codons, we hypothesized that codon usage may correlate with Pab1 occupancy and have consequences on deadenylation.

To examine this, we analyzed data from a recently published Pab1 RNA-immunoprecipitation experiment (Costello et al., 2015). We binned mRNAs by codon optimality and asked whether there were differences in Pab1 binding per length of poly(A) tail (Subtelny et al., 2014). Strikingly, we observed that when normalized to overall mRNA poly(A) tail length, mRNAs of high codon optimality had enhanced Pab1 association compared to those of low codon optimality (Figure 5A). These data reveal a distinction in mRNP composition that is correlated with codon optimality and thus with translational elongation rate.

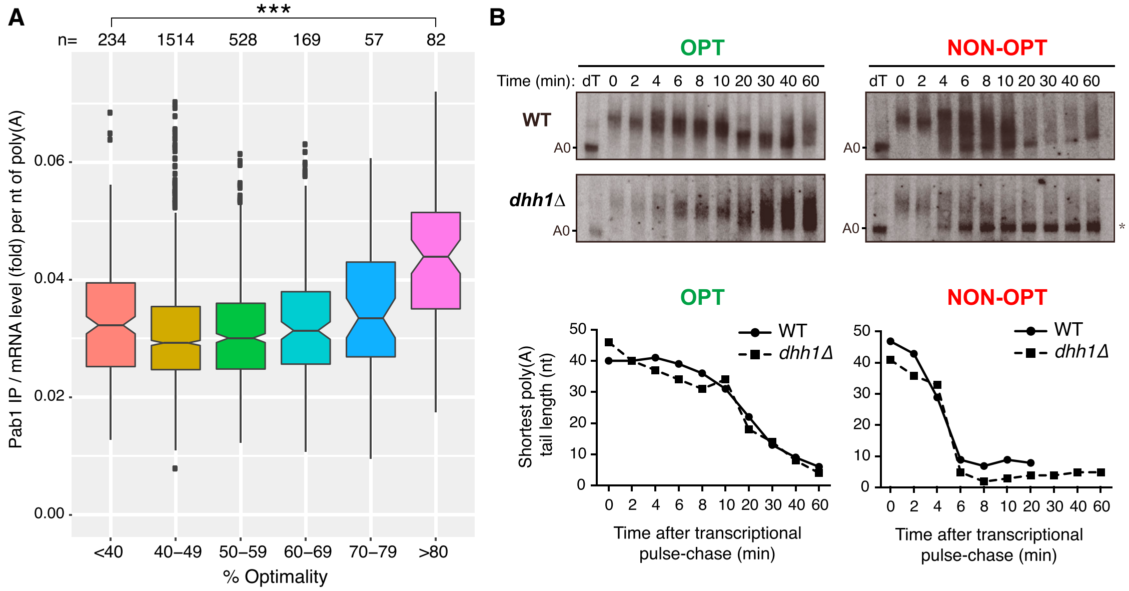

Figure 5. Codon Optimality Influences Pab1 Association and mRNA Deadenylation Rate

(A) Plot of Pab1-bound mRNA levels relative to total mRNA levels following normalization to poly(A) tail length and binning of mRNAs according to codon optimality. Values were calculated using previously published Pab1 RNA immunoprecipitation sequencing (RIP-seq), total RNA sequencing (RNA-seq), and poly(A) tail length profiling by sequencing (PAL-seq) data. ***$p_{adj} < 10^{-3}$.

(B) High-resolution polyacrylamide northern blots and plots of shortest poly(A) tail lengths of the OPT and NON-OPT mRNAs following *GAL1* transcriptional pulse-chase experiments in WT or *dhh1Δ* cells. A0 indicates the migration of a completely deadenylated mRNA species. Asterisk denotes the accumulation of deadenylated mRNA species. The lane labeled dT is the 0 time point treated with oligo dT and RNaseH to indicate the migration position of fully deadenylated mRNA. Representative gels and plots of experiments done in triplicate are shown.

Codon Optimality Influences mRNA Deadenylation Rate

Differences in Pab1 occupancy might contribute to altered patterns of deadenylation between optimal and non-optimal transcripts. To test this, we utilized reporter constructs encoding mRNA transcripts that are identical in UTR composition and encode the exact same polypeptide but represent extremes of overall codon optimality. The OPT mRNA contains only optimal codons, while the NON-OPT mRNA bears only non-optimal codons; this distinction causes them to degrade with a 4-fold difference in half-life (Presnyak et al., 2015). Poly(A) tail lengths were visualized by northern blot after transcriptional pulse chase in *S. cerevisiae*.

In wild-type cells, the OPT mRNA was deadenylated more slowly than the NON-OPT mRNA, with oligo(A) species (<A15) observed 30 min and 6 min, respectively, after inhibition of reporter transcription (Figure 5B). Deadenylation patterns are therefore sensitive to codon optimality.

We recently showed that the decapping activator Dhh1/DDX6 is critical to coupling low codon optimality to rapid mRNA decay (Radhakrishnan et al., 2016). To test whether differences in deadenylation of OPT and NON-OPT mRNAs depend on Dhh1, we compared deadenylation of the reporter RNAs in wild-type and *dhh1Δ* cells (Figure 5B). Deadenylation profiles were similar to wild-type, indicating that Dhh1 is not required for differences in deadenylation. The loss of Dhh1 function does, however, result in the accumulation of deadenylated mRNA that is likely stable because it does not undergo efficient decapping (Figure 5B, asterisk) (Coller and Parker, 2005). These results show that codon optimality impacts deadenylation upstream and independent of Dhh1.

Codon Optimality Differentiates the Roles of Ccr4 and Caf1 in Deadenylation

Given that Pab1 occupancy is correlated with codon optimality and that Pab1 differentiates the two nucleases of Ccr4-Not, we analyzed the roles of Ccr4 and Caf1 in deadenylation of the reporter constructs with optimal and non-optimal codons after transcriptional shut-off *in vivo*. Deletion of *CCR4* stabilized both OPT and NON-OPT mRNAs (2.7-fold and 5.2-fold relative to WT, respectively; Figure 6A). This indicates that Ccr4 acts as a general deadenylase whose function in mRNA decay is independent of the influence of codon optimality. This is consistent with our finding that Ccr4 can remove poly(A) tails independent of whether Pab1 is bound.

In contrast, deletion of *CAF1* increased the stability of NON-OPT mRNA (3.1-fold relative to WT) but did not substantially affect the stability of OPT mRNA (Figure 6A). Similar selectivity of Caf1, but not Ccr4, for non-optimal mRNAs was observed using a distinct set of reporters varying in codon optimality (Figures S7A–S7C). These data indicate that Caf1 preferentially destabilizes non-optimal mRNAs. We propose that Pab1 enrichment on highly optimal mRNAs prevents Caf1 from contributing to their deadenylation, while lower levels of Pab1 on low-optimality mRNAs permits Caf1 function.

To determine whether Caf1-dependent decay of non-optimal mRNAs was due to more rapid deadenylation, we analyzed poly(A) tail lengths in pulse-chase experiments. The rapid

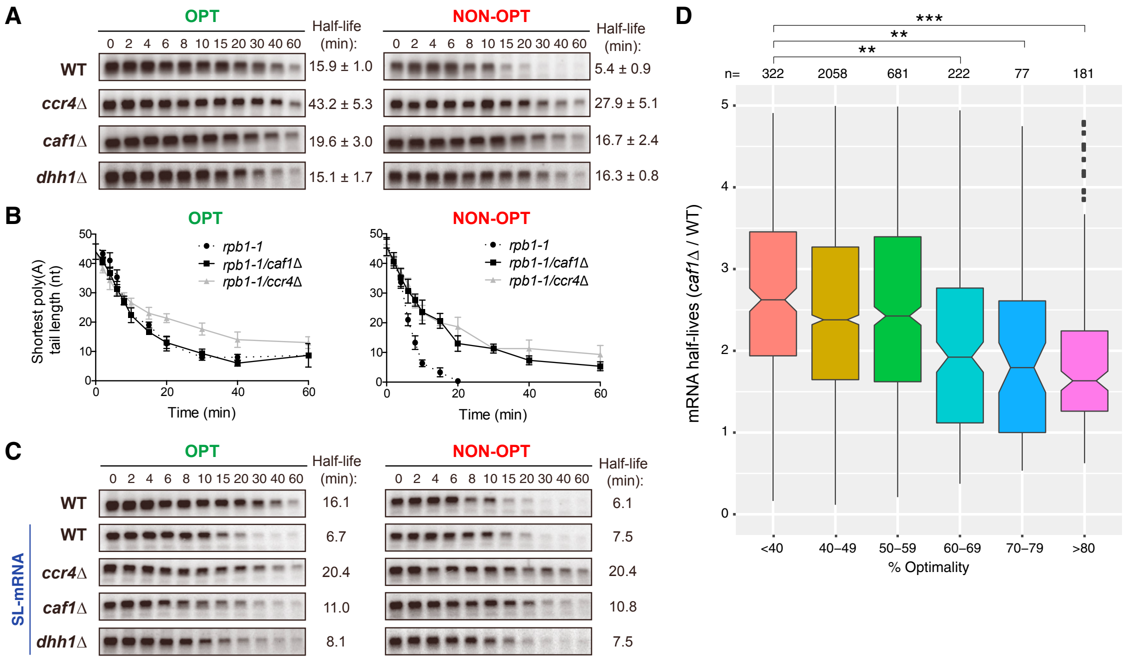

Figure 6. Caf1 Destabilizes mRNAs with Low Codon Optimality by Accelerating Deadenylation Rate

(A) Northern blots of the OPT and NON-OPT reporters following *GAL1* transcriptional shut-off experiments in WT, *ccr4*Δ, *caf1*Δ, and *dhh1*Δ yeast. mRNA half-lives are represented as mean ± standard deviation for experiments performed with four (*dhh1*Δ, *ccr4*Δ) or five (WT, *caf1*Δ) replicates.

(B) Plots showing the deadenylation rate of the OPT and NON-OPT reporters in *rpb1-1*, *rpb1-1/ccr4*Δ, or *rpb1-1/caf1*Δ yeast determined from transcriptional pulse-chase experiments (see Figure S7). Data points are represented as mean ± standard deviations for experiments performed in triplicate.

(C) Northern blots of OPT and NON-OPT reporters in WT yeast and OPT and NON-OPT reporters containing a stem loop (SL) in the 5′ UTR (SL-mRNA) in WT, *ccr4*Δ, *caf1*Δ, or *dhh1*Δ yeast after *GAL1* transcriptional shut-off experiments.

(D) Plot of *S. cerevisiae* mRNA half-lives in *caf1*Δ cells relative to WT cells binned according to codon optimality. **: $10^{-2} > p_{adj} > 10^{-3}$; ***$p_{adj} < 10^{-3}$.

See also Figure S7 and Table S1.

deadenylation of NON-OPT mRNA in wild-type cells was abolished by deletion of *CAF1* (Figures 6B and S7D; Table S1). Importantly, the NON-OPT mRNA was deadenylated at a similar rate as the OPT mRNA in cells lacking *CAF1*. Therefore, Caf1 plays an important role in discriminating between optimal and non-optimal mRNAs by selectively accelerating deadenylation of non-optimal mRNAs.

Deletion of *CCR4* resulted in an ~3.3- or 4.7-fold decrease of the deadenylation rate for both OPT and NON-OPT mRNAs, respectively (Figure 6B; Table S1). This is consistent with its ability to remove all poly(A) tails independent of the presence of Pab1.

Caf1 Regulates mRNA Decay in a Translation-Dependent Manner

Preferential destabilization of non-optimal mRNAs by Caf1 is reminiscent of Dhh1 activity (Figure 6A). Since Dhh1 communicates translational elongation rate to mRNA decapping, its decay function requires mRNA translation (Coller and Parker, 2005). To test whether the role of Caf1 in mRNA decay is also translation dependent, we introduced a stem-loop (SL) secondary structure into the 5′ UTR of reporter mRNAs (SL-OPT and SL-NON-OPT mRNAs) to inhibit translation. The SL structure has been shown to limit 48S ribosome scanning and reduce protein production to less than 10% (Beelman and Parker, 1994; Sweet et al., 2012).

When translation of reporter mRNAs was blocked by the SL structure in WT cells, a difference in the half-lives of OPT and NON-OPT mRNAs was no longer observed (Figure 6C). This is consistent with our previous finding that translation is central to differential mRNA stability (Presnyak et al., 2015).

We next performed transcriptional shut-off experiments on SL-containing OPT and NON-OPT mRNAs in cells lacking Caf1, Ccr4, or Dhh1. The SL-NON-OPT mRNA was not substantially stabilized in *caf1*Δ or *dhh1*Δ cells (Figure 6C). Furthermore, the half-lives of SL-OPT and SL-NON-OPT mRNAs are indistinguishable in these strains. In contrast, both SL-OPT and SL-NON-OPT mRNAs were stabilized in *ccr4*Δ cells (Figure 6C). This indicates that translation is not required for Ccr4 function. These results show that, like Dhh1, Caf1 discriminates between mRNAs of different codon optimality and regulates mRNA degradation in a translation-dependent manner, but Ccr4 is a general deadenylase affecting degradation of all mRNAs.

Loss of *CAF1* Broadly Stabilizes mRNAs of Low Codon Optimality

To determine whether Caf1 has a broad influence on the decay of mRNAs as a function of codon optimality, we conducted a genomic transcriptional shut-off experiment using a temperature-sensitive allele of RNA polymerase II (Pol II). Following

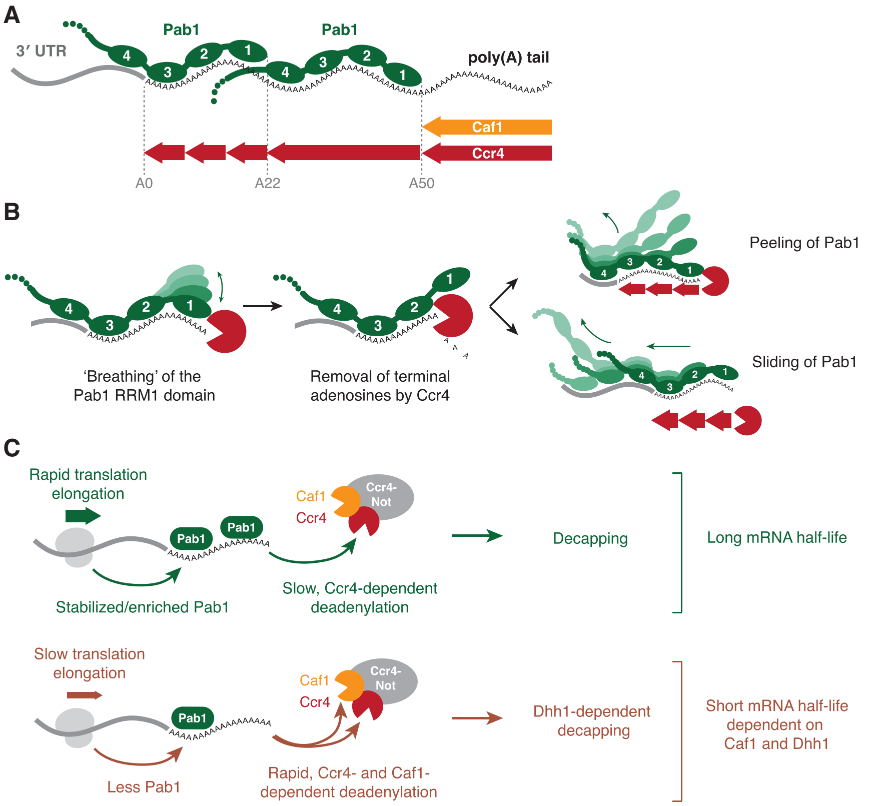

Figure 7. Models for Pab1 Release by Ccr4 and Coupling of Translation and Deadenylation Rates by Caf1

(A) Proposed model for the organization of Pab1 on the poly(A) tail with RRMs depicted linearly. The Pab1 molecule proximal to the 3′ UTR binds ~22 adenosines through RRMs 1–3, and distal Pab1 molecules bind ~28 adenosines with RRMs 1–4. Naked poly(A) not bound by Pab1 can be removed by either Caf1 or Ccr4, while RNA within the binding site of Pab1 can only be accessed by Ccr4. Pab1 self-association and interaction with other proteins may lead to higher-order structures on RNA.

(B) The modular architecture of Pab1 permits deadenylation to occur before it completely dissociates from the poly(A) tail.

(C) Translation elongation rate may contribute to Pab1 occupancy to affect deadenylation rate. Ccr4 is required for deadenylation of all mRNAs, but the requirement for Caf1 is specific to mRNAs with low codon optimality or reduced Pab1 occupancy.

heat inactivation of Pol II in *rpb1-1* and *rpb1-1/caf1*Δ strains, cells were harvested at various time points, and then global mRNA decay analysis was performed by RNA sequencing (RNA-seq) on libraries from each time point. We have used this approach successfully in the past and have shown that our data correlate well with mRNA half-lives achieved by other methods, such as metabolic labeling approaches (Presnyak et al., 2015).

Using this approach, we obtained reproducible half-lives for 3,535 mRNAs in budding yeast. Binning mRNAs by codon optimality demonstrated that loss of *CAF1* generally stabilizes mRNAs of low codon optimality (Figure 6D). Taken together, our data suggest that deadenylation rate is enhanced on mRNAs of low codon optimality via the concerted efforts of Ccr4 and Caf1, while high-optimality transcripts are subject to only Ccr4-mediated deadenylation.

DISCUSSION

Deadenylation is a widespread process that regulates the translation and stability of eukaryotic mRNAs. While involvement of the Ccr4-Not complex and Pab1/PABPC1 in this process is well established, the way in which they directly affect each other and are influenced by other cellular signals has remained elusive. Here, we provide mechanistic insight into this process, showing that Pab1 does not block Ccr4-Not nuclease activity and that Pab1 can be efficiently released from RNAs by Ccr4, but not Caf1 (Figure 7A). The two nucleases are differentially active on low and high codon optimality transcripts. We propose that this is a result of differential Pab1 occupancy.

A Model for mRNA Deadenylation

Previous data suggested that Pan2-Pan3 may initiate deadenylation in cells whereas Ccr4-Not more efficiently removes shorter poly(A) tails (Tucker et al., 2001; Yamashita et al., 2005). This is consistent with a model of deadenylation in which these complexes act sequentially. It was proposed that the mechanistic basis for these two phases of deadenylation is binding of Pab1 to the poly(A) tail: Pan2-Pan3 is stimulated by Pab1 and would therefore remove the distal portion of the (Pab1-bound) poly(A) tail, while subsequent shortening of the proximal poly(A) tail by Ccr4-Not would only occur once Pab1 had been displaced (Tucker et al., 2001; Yamashita et al., 2005). Our data showing that Pab1 can be efficiently released *in vitro* by the exonuclease activity of Ccr4-Not suggest that Pab1 binding alone does not provide a mechanistic explanation for sequential deadenylation. In fact, deadenylation of Pab1-bound A60 RNAs by Ccr4-Not is faster than deadenylation on RNAs without Pab1.

In vivo, Ccr4 compensates for deletion of Pan2 (Tucker et al., 2001). In contrast, deadenylation does not proceed beyond ~22 adenosines in the absence of Ccr4 (Figure 2B). This corresponds in size to the footprint of one Pab1 and suggests that Ccr4-Not, but not Pan2-Pan3, is able to efficiently release the final molecule of Pab1 from the poly(A) tail *in vivo*. Thus, our data are consistent with a model in which either complex can initiate deadenylation but only Ccr4-Not can remove the proximal part of the poly(A) tail.

Release of the final Pab1 precedes mRNA decapping and decay and is likely a critical point of regulation. Recent data suggest that highly expressed eukaryotic mRNAs often have short poly(A) tails (20–30 adenosines) (Lima et al., 2017).

Furthermore, translation efficiency in somatic cells decreases only when the poly(A) tail is shortened to less than 30 adenosines (Park et al., 2016; Subtelny et al., 2014). Combined, these data suggest that only one Pab1/PABPC1 molecule is required for efficient translation and high stability of mRNAs. We show here that the UTR-proximal Pab1 molecule binds RNA in a different manner to Pab1 on distal parts of the poly(A) tail: RRM 4 is not specific for poly(A), allowing the molecule to bridge the 3′ UTR:poly(A) tail junction. It is likely that Ccr4-Not plays a crucial role in releasing this final, 3′ UTR-proximal Pab1 molecule.

Mechanism of Pab1 Release

Deadenylation by Ccr4-Not proceeds in a stepwise pattern, likely because RRMs 1, 2, and 3 of Pab1 each protect ~8 adenosines, and Ccr4-Not stalls when it encounters each RRM. If the terminal (3′) RRM disengages from the RNA (due to "breathing"), Ccr4-Not can remove terminal ribonucleotides, preventing the RRM from rebinding and resulting in tail shortening until another RRM is encountered (Figure 7B). This process is unidirectional, because once the tail is shortened, the RRM is unable to rebind. Each subsequent RRM is removed in the same manner. This could result in "peeling" of Pab1 off the RNA, one RRM at a time. Alternatively, Pab1 could slide or ratchet along the RNA: the 5′ proximal Pab1 molecule would move further into the 3′ UTR, allowing RRMs 1 and 2 to retain their high-affinity interactions with poly(A) RNA. The final Pab1 will be released once the tail is shorter than 8 nucleotides and no RRMs contact poly(A).

Each RRM domain of Pab1 binds RNA with low affinity (Burd et al., 1991; Kühn and Pieler, 1996; Nietfeld et al., 1990), but avidity effects increase overall affinity. Thus, the modular architecture of Pab1 allows shortening of the poly(A) tail without Pab1 completely dissociating from the mRNA. These are important properties for a protein that must remain stably bound to mRNA to promote translation and prevent decapping but that can also be rapidly released to promote translation repression and mRNA decay in response to cellular signals.

The Pab1-Bound Poly(A) Tail Is Shortened by Ccr4, but Not Caf1

Although both Ccr4 and Caf1 are poly(A)-selective exonucleases, Pab1 differentiates their activities, and only Ccr4 is active on RNA bound by Pab1 (Figure 7A). Ccr4 is thought to be the major deadenylase in *S. cerevisiae* (Thore et al., 2003; Tucker et al., 2001, 2002; Balagopal et al., 2017). Based on our findings, this is likely due to the requirement for Ccr4 on Pab1-bound poly(A) tails.

Pab1 stimulates Ccr4 both in the Ccr4-Not complex and as an isolated protein, at least, in part, through direct contact with the EEP nuclease domain. Other mRNA-binding proteins interact with specific sequences in the 3′ UTR, and these are required for efficient deadenylation of specific transcripts. Compared to these other mRNA-binding proteins that recruit Ccr4-Not, the interaction between Pab1 and Ccr4-Not is likely lower affinity. By binding Pab1, Ccr4-Not may be able to efficiently locate the 3′ terminus of the mRNA to permit exonucleolytic activity.

Ccr4-Not and Pab1 Couple Translation with mRNA Decay

Our analysis reveals that the abundance or stability of Pab1 on the poly(A) tail correlates with the translation elongation rate set by codon optimality. Furthermore, Pab1 and the nucleases of Ccr4-Not provide a mechanistic link between the processes of mRNA decay and translation. Ccr4 shortens poly(A) independent of the presence of Pab1 and is a general deadenylase acting on all mRNAs. In contrast, Caf1 only shortens poly(A) that is not bound by Pab1 and is selective for non-optimal transcripts that are associated with lower Pab1 occupancy (Figure 7C). We previously reported that the decapping activator Dhh1/DDX6 is required for sensing and rapidly degrading mRNAs with low codon optimality. We show here that Caf1 is similarly essential to this process, acting upstream of Dhh1.

A critical and unresolved question is how translational elongation rates influence the overall mRNP architecture, including Pab1 binding, and how this makes transcripts differentially susceptible to deadenylases and Dhh1. There is a long-standing appreciation that translation rates and mRNA decay rates are tightly coupled. Our work provides a mechanistic understanding of the emerging concept that the mRNA degradation machinery is highly orchestrated to monitor the translatability of mRNAs.

STAR★METHODS

Detailed methods are provided in the online version of this paper and include the following:

- KEY RESOURCES TABLE
- CONTACT FOR REAGENT AND RESOURCE SHARING
- EXPERIMENTAL MODEL AND SUBJECT DETAILS
- METHOD DETAILS
  - Protein Purification
  - Deadenylation Assays
  - Electrophoretic Mobility Shift Assays
  - Fluorescence Polarization Assays
  - SwitchSENSE Kinetic Analysis
  - Yeast Strains and Growth Conditions
  - RNA Labeling and RNase A/T1 Digestion
  - Reporter Construction
  - Transcriptional Shut-Off and Pulse-Chase
  - Global mRNA Half-Life Analysis
  - Analysis of Pab1 Occupancy
- QUANTIFICATION AND STATISTICAL ANALYSIS
  - *In Vitro* Deadenylation Analysis
  - Fluorescence Polarization Assays
  - SwitchSENSE Kinetic Analysis
  - *In Vivo* Deadenylation Analysis
  - *In Vivo* mRNA Half-Life Analysis
  - Pab1 Enrichment
- DATA AND SOFTWARE AVAILABILITY

SUPPLEMENTAL INFORMATION

Supplemental Information includes seven figures and two tables and can be found with this article online at https://doi.org/10.1016/j.molcel.2018.05.033.

ACKNOWLEDGMENTS

We thank Ramanujan Hegde, Terence Tang, and Vish Chandrasekara for comments on the manuscript; Jana Wolf for contributions to the project in the early stages; and Stephen McLaughlin and Chris Johnson for assistance and advice with biophysical techniques. This work was supported by the European Research Council under the European Union's Seventh Framework Programme (FP7/2007-2013)/ERC starting grant No. 261151 (to L.A.P.); under the European Union's Horizon 2020 research and innovation programme (ERC Consolidator grant agreement No. 725685) (to L.A.P.); Medical Research Council (MRC) grant MC_U105192715 (L.A.P,); and NIH grants GM118018 (J.C.), GM125086 (J.C.), and GM118140 (B.R.G.).

AUTHOR CONTRIBUTIONS

M.W.W. and J.A.W.S. purified proteins; M.W.W. performed binding experiments and activity assays; M.W.W., J.A.W.S., and L.A.P. designed experiments and interpreted data; Y.-H.C., N.A., T.S., and J.C. performed *in vivo* yeast experiments; B.R.G. performed RNA-seq; L.A.P. and J.C. supervised the project; M.W.W., J.C., and L.A.P. wrote the manuscript with contributions from all authors.

DECLARATION OF INTERESTS

The authors declare no competing interests.

Received: February 27, 2018
Revised: May 17, 2018
Accepted: May 24, 2018
Published: June 21, 2018

SUPPORTING CITATIONS

The following references appear in the Supplemental Information: Jones and Cozzetto (2015); Yao et al. (2007).

REFERENCES

Baejen, C., Torkler, P., Gressel, S., Essig, K., Söding, J., and Cramer, P. (2014). Transcriptome maps of mRNP biogenesis factors define pre-mRNA recognition. Mol. Cell *55*, 745–757.

Baer, B.W., and Kornberg, R.D. (1983). The protein responsible for the repeating structure of cytoplasmic poly(A)-ribonucleoprotein. J. Cell Biol. *96*, 717–721.

Balagopal, V., Bolisetty, M., Al Husaini, N., Coller, J., and Graveley, B.R. (2017). Ccr4 and Pop2 control poly(A) tail length in *Saccharomyces cerevisiae*. bioRxiv. https://doi.org/10.1101/140202.

Bazzini, A.A., Del Viso, F., Moreno-Mateos, M.A., Johnstone, T.G., Vejnar, C.E., Qin, Y., Yao, J., Khokha, M.K., and Giraldez, A.J. (2016). Codon identity regulates mRNA stability and translation efficiency during the maternal-to-zygotic transition. EMBO J. *35*, 2087–2103.

Beelman, C.A., and Parker, R. (1994). Differential effects of translational inhibition in cis and in trans on the decay of the unstable yeast MFA2 mRNA. J. Biol. Chem. *269*, 9687–9692.

Belloc, E., and Méndez, R. (2008). A deadenylation negative feedback mechanism governs meiotic metaphase arrest. Nature *452*, 1017–1021.

Bianchin, C., Mauxion, F., Sentis, S., Séraphin, B., and Corbo, L. (2005). Conservation of the deadenylase activity of proteins of the Caf1 family in human. RNA *11*, 487–494.

Burd, C.G., Matunis, E.L., and Dreyfuss, G. (1991). The multiple RNA-binding domains of the mRNA poly(A)-binding protein have different RNA-binding activities. Mol. Cell. Biol. *11*, 3419–3424.

Caponigro, G., and Parker, R. (1995). Multiple functions for the poly(A)-binding protein in mRNA decapping and deadenylation in yeast. Genes Dev. *9*, 2421–2432.

Carballo, E., Lai, W.S., and Blackshear, P.J. (1998). Feedback inhibition of macrophage tumor necrosis factor-alpha production by tristetraprolin. Science *281*, 1001–1005.

Cheng, J., Maier, K.C., Avsec, Ž., Rus, P., and Gagneur, J. (2017). *Cis*-regulatory elements explain most of the mRNA stability variation across genes in yeast. RNA *23*, 1648–1659.

Cléry, A., Sohier, T.J.M., Welte, T., Langer, A., and Allain, F.H.T. (2017). switchSENSE: a new technology to study protein-RNA interactions. Methods *118-119*, 137–145.

Coller, J., and Parker, R. (2005). General translational repression by activators of mRNA decapping. Cell *122*, 875–886.

Coller, J.M., Gray, N.K., and Wickens, M.P. (1998). mRNA stabilization by poly(A) binding protein is independent of poly(A) and requires translation. Genes Dev. *12*, 3226–3235.

Costello, J., Castelli, L.M., Rowe, W., Kershaw, C.J., Talavera, D., Mohammad-Qureshi, S.S., Sims, P.F.G., Grant, C.M., Pavitt, G.D., Hubbard, S.J., and Ashe, M.P. (2015). Global mRNA selection mechanisms for translation initiation. Genome Biol. *16*, 10.

Daugeron, M.C., Mauxion, F., and Séraphin, B. (2001). The yeast POP2 gene encodes a nuclease involved in mRNA deadenylation. Nucleic Acids Res. *29*, 2448–2455.

Deardorff, J.A., and Sachs, A.B. (1997). Differential effects of aromatic and charged residue substitutions in the RNA binding domains of the yeast poly(A)-binding protein. J. Mol. Biol. *269*, 67–81.

Fabian, M.R., Mathonnet, G., Sundermeier, T., Mathys, H., Zipprich, J.T., Svitkin, Y.V., Rivas, F., Jinek, M., Wohlschlegel, J., Doudna, J.A., et al. (2009). Mammalian miRNA RISC recruits CAF1 and PABP to affect PABP-dependent deadenylation. Mol. Cell *35*, 868–880.

Geisler, S., Lojek, L., Khalil, A.M., Baker, K.E., and Coller, J. (2012). Decapping of long noncoding RNAs regulates inducible genes. Mol. Cell *45*, 279–291.

Goldstrohm, A.C., Hook, B.A., Seay, D.J., and Wickens, M. (2006). PUF proteins bind Pop2p to regulate messenger RNAs. Nat. Struct. Mol. Biol. *13*, 533–539.

Jones, D.T., and Cozzetto, D. (2015). DISOPRED3: precise disordered region predictions with annotated protein-binding activity. Bioinformatics *31*, 857–863.

Khaleghpour, K., Svitkin, Y.V., Craig, A.W., DeMaria, C.T., Deo, R.C., Burley, S.K., and Sonenberg, N. (2001). Translational repression by a novel partner of human poly(A) binding protein, Paip2. Mol. Cell *7*, 205–216.

Kramer, K., Sachsenberg, T., Beckmann, B.M., Qamar, S., Boon, K.-L., Hentze, M.W., Kohlbacher, O., and Urlaub, H. (2014). Photo-cross-linking and high-resolution mass spectrometry for assignment of RNA-binding sites in RNA-binding proteins. Nat. Methods *11*, 1064–1070.

Kühn, U., and Pieler, T. (1996). Xenopus poly(A) binding protein: functional domains in RNA binding and protein-protein interaction. J. Mol. Biol. *256*, 20–30.

Langer, A., Hampel, P.A., Kaiser, W., Knezevic, J., Welte, T., Villa, V., Maruyama, M., Svejda, M., Jähner, S., Fischer, F., et al. (2013). Protein analysis by time-resolved measurements with an electro-switchable DNA chip. Nat. Commun. *4*, 2099.

Langmead, B., Trapnell, C., Pop, M., and Salzberg, S.L. (2009). Ultrafast and memory-efficient alignment of short DNA sequences to the human genome. Genome Biol. *10*, R25.

Lee, D., Ohn, T., Chiang, Y.-C., Quigley, G., Yao, G., Liu, Y., and Denis, C.L. (2010). PUF3 acceleration of deadenylation in vivo can operate independently of CCR4 activity, possibly involving effects on the PAB1-mRNP structure. J. Mol. Biol. *399*, 562–575.

Li, H., Handsaker, B., Wysoker, A., Fennell, T., Ruan, J., Homer, N., Marth, G., Abecasis, G., and Durbin, R.; 1000 Genome Project Data Processing Subgroup (2009). The Sequence Alignment/Map format and SAMtools. Bioinformatics *25*, 2078–2079.

Lima, S.A., Chipman, L.B., Nicholson, A.L., Chen, Y.-H., Yee, B.A., Yeo, G.W., Coller, J., and Pasquinelli, A.E. (2017). Short poly(A) tails are a conserved feature of highly expressed genes. Nat. Struct. Mol. Biol. *24*, 1057–1063.

Mangus, D.A., Evans, M.C., and Jacobson, A. (2003). Poly(A)-binding proteins: multifunctional scaffolds for the post-transcriptional control of gene expression. Genome Biol. *4*, 223.

Mishima, Y., and Tomari, Y. (2016). Codon usage and 3′ UTR length determine maternal mRNA stability in zebrafish. Mol. Cell *61*, 874–885.

Nietfeld, W., Mentzel, H., and Pieler, T. (1990). The Xenopus laevis poly(A) binding protein is composed of multiple functionally independent RNA binding domains. EMBO J. *9*, 3699–3705.

Nonet, M., Scafe, C., Sexton, J., and Young, R. (1987). Eucaryotic RNA polymerase conditional mutant that rapidly ceases mRNA synthesis. Mol. Cell. Biol. *7*, 1602–1611.

Park, J.-E., Yi, H., Kim, Y., Chang, H., and Kim, V.N. (2016). Regulation of poly(A) tail and translation during the somatic cell cycle. Mol. Cell *62*, 462–471.

Parker, R. (2012). RNA degradation in Saccharomyces cerevisae. Genetics *191*, 671–702.

Pechmann, S., and Frydman, J. (2013). Evolutionary conservation of codon optimality reveals hidden signatures of cotranslational folding. Nat. Struct. Mol. Biol. *20*, 237–243.

Presnyak, V., Alhusaini, N., Chen, Y.-H., Martin, S., Morris, N., Kline, N., Olson, S., Weinberg, D., Baker, K.E., Graveley, B.R., and Coller, J. (2015). Codon optimality is a major determinant of mRNA stability. Cell *160*, 1111–1124.

Radhakrishnan, A., Chen, Y.-H., Martin, S., Alhusaini, N., Green, R., and Coller, J. (2016). The DEAD-box protein Dhh1p couples mRNA decay and translation by monitoring codon optimality. Cell *167*, 122–132.e9.

Rissland, O.S., Subtelny, A.O., Wang, M., Lugowski, A., Nicholson, B., Laver, J.D., Sidhu, S.S., Smibert, C.A., Lipshitz, H.D., and Bartel, D.P. (2017). The influence of microRNAs and poly(A) tail length on endogenous mRNA-protein complexes. Genome Biol. *18*, 211.

Sachs, A.B., and Davis, R.W. (1989). The poly(A) binding protein is required for poly(A) shortening and 60S ribosomal subunit-dependent translation initiation. Cell *58*, 857–867.

Sachs, A.B., Davis, R.W., and Kornberg, R.D. (1987). A single domain of yeast poly(A)-binding protein is necessary and sufficient for RNA binding and cell viability. Mol. Cell. Biol. *7*, 3268–3276.

Schneider, C.A., Rasband, W.S., and Eliceiri, K.W. (2012). NIH Image to ImageJ: 25 years of image analysis. Nat. Methods *9*, 671–675.

Simón, E., and Séraphin, B. (2007). A specific role for the C-terminal region of the Poly(A)-binding protein in mRNA decay. Nucleic Acids Res. *35*, 6017–6028.

Stowell, J.A.W., Webster, M.W., Kögel, A., Wolf, J., Shelley, K.L., and Passmore, L.A. (2016). Reconstitution of targeted deadenylation by the Ccr4-Not complex and the YTH domain protein Mmi1. Cell Rep. *17*, 1978–1989.

Subtelny, A.O., Eichhorn, S.W., Chen, G.R., Sive, H., and Bartel, D.P. (2014). Poly(A)-tail profiling reveals an embryonic switch in translational control. Nature *508*, 66–71.

Sweet, T., Kovalak, C., and Coller, J. (2012). The DEAD-box protein Dhh1 promotes decapping by slowing ribosome movement. PLoS Biol. *10*, e1001342.

Thore, S., Mauxion, F., Séraphin, B., and Suck, D. (2003). X-ray structure and activity of the yeast Pop2 protein: a nuclease subunit of the mRNA deadenylase complex. EMBO Rep. *4*, 1150–1155.

Trapnell, C., Williams, B.A., Pertea, G., Mortazavi, A., Kwan, G., van Baren, M.J., Salzberg, S.L., Wold, B.J., and Pachter, L. (2010). Transcript assembly and quantification by RNA-Seq reveals unannotated transcripts and isoform switching during cell differentiation. Nat. Biotechnol. *28*, 511–515.

Tuck, A.C., and Tollervey, D. (2013). A transcriptome-wide atlas of RNP composition reveals diverse classes of mRNAs and lncRNAs. Cell *154*, 996–1009.

Tucker, M., Valencia-Sanchez, M.A., Staples, R.R., Chen, J., Denis, C.L., and Parker, R. (2001). The transcription factor associated Ccr4 and Caf1 proteins are components of the major cytoplasmic mRNA deadenylase in Saccharomyces cerevisiae. Cell *104*, 377–386.

Tucker, M., Staples, R.R., Valencia-Sanchez, M.A., Muhlrad, D., and Parker, R. (2002). Ccr4p is the catalytic subunit of a Ccr4p/Pop2p/Notp mRNA deadenylase complex in Saccharomyces cerevisiae. EMBO J. *21*, 1427–1436.

Viswanathan, P., Ohn, T., Chiang, Y.-C., Chen, J., and Denis, C.L. (2004). Mouse CAF1 can function as a processive deadenylase/3′-5′-exonuclease in vitro but in yeast the deadenylase function of CAF1 is not required for mRNA poly(A) removal. J. Biol. Chem. *279*, 23988–23995.

Wahle, E., and Winkler, G.S. (2013). RNA decay machines: deadenylation by the Ccr4-not and Pan2-Pan3 complexes. Biochim. Biophys. Acta *1829*, 561–570.

Wang, H., Morita, M., Yang, X., Suzuki, T., Yang, W., Wang, J., Ito, K., Wang, Q., Zhao, C., Bartlam, M., et al. (2010). Crystal structure of the human CNOT6L nuclease domain reveals strict poly(A) substrate specificity. EMBO J. *29*, 2566–2576.

Webster, M.W., Stowell, J.A.W., Tang, T.T.L., and Passmore, L.A. (2017). Analysis of mRNA deadenylation by multi-protein complexes. Methods *126*, 95–104.

Weidmann, C.A., Raynard, N.A., Blewett, N.H., Van Etten, J., and Goldstrohm, A.C. (2014). The RNA binding domain of Pumilio antagonizes poly-adenosine binding protein and accelerates deadenylation. RNA *20*, 1298–1319.

Wolf, J., Valkov, E., Allen, M.D., Meineke, B., Gordiyenko, Y., McLaughlin, S.H., Olsen, T.M., Robinson, C.V., Bycroft, M., Stewart, M., and Passmore, L.A. (2014). Structural basis for Pan3 binding to Pan2 and its function in mRNA recruitment and deadenylation. EMBO J. *33*, 1514–1526.

Yamashita, A., Chang, T.-C., Yamashita, Y., Zhu, W., Zhong, Z., Chen, C.-Y.A., and Shyu, A.-B. (2005). Concerted action of poly(A) nucleases and decapping enzyme in mammalian mRNA turnover. Nat. Struct. Mol. Biol. *12*, 1054–1063.

Yao, G., Chiang, Y.-C., Zhang, C., Lee, D.J., Laue, T.M., and Denis, C.L. (2007). PAB1 self-association precludes its binding to poly(A), thereby accelerating CCR4 deadenylation in vivo. Mol. Cell. Biol. *27*, 6243–6253.

Zekri, L., Kuzuoğlu-Öztürk, D., and Izaurralde, E. (2013). GW182 proteins cause PABP dissociation from silenced miRNA targets in the absence of deadenylation. EMBO J. *32*, 1052–1065.

STAR★METHODS

KEY RESOURCES TABLE

REAGENT or RESOURCE	SOURCE	IDENTIFIER
Bacterial and Virus Strains		
E. coli BL21 star (DE3)	Thermo Fisher Scientific	C601003
E. coli DH5α (DE3)	Thermo Fisher Scientific	18258012
Chemicals, Peptides, and Recombinant Proteins		
Insect-XPRESS protein-free insect cell medium with L-glutamine	Lonza	12-730Q
Protease Inhibitor Cocktail	Sigma-Aldrich	11836170001
Desthiobiotin	IBA	2-1000-001
Imidazole	Sigma-Aldrich	I5513
Formamide	Sigma-Aldrich	11814320001
TEMED	Sigma-Aldrich	T9281
Ammonium persulfate (APS)	Sigma-Aldrich	A3678
SYBR Safe DNA Gel Stain	Thermo Fisher Scientific	S33102
SYBR Green II RNA Gel Stain	Thermo Fisher Scientific	S7586
Ni-NTA Agarose	QIAGEN	30210
Glutathione Sepharose 4B	GE Healthcare	17075601
Amicon Ultra Centrifugal Filter Units	Millipore	UFC901096
TWEEN 20	Sigma-Aldrich	P9416
S. pombe Ccr4-Not (WT) protein	Stowell et al., 2016	N/A
S. pombe Ccr4-Not (Ccr4 E387A) protein	Stowell et al., 2016	N/A
S. pombe Ccr4-Not (Caf1 D53A) protein	Stowell et al., 2016	N/A
S. pombe Ccr4-Caf1 (WT) protein	This paper	N/A
S. pombe Ccr4-Caf1 (Ccr4 E387A) protein	This paper	N/A
S. pombe Ccr4-Caf1 (Caf1 D53A) protein	This paper	N/A
S. pombe Ccr4 (EEP domain) protein	This paper	N/A
S. pombe Caf1 protein	This paper	N/A
S. pombe Pab1 protein	This paper	N/A
S. pombe Pab1ΔC protein	This paper	N/A
S. pombe Pab1ΔPC protein	This paper	N/A
S. pombe Pab1 (Y83A) protein	This paper	N/A
S. pombe Pab1 (F171A) protein	This paper	N/A
S. pombe Pab1 (Y264A) protein	This paper	N/A
S. pombe Pab1 (F367A) protein	This paper	N/A
Critical Commercial Assays		
Quikchange Lightning Multi Site-Directed Mutagenesis Kit	Agilent Technologies	210513
In-Fusion HD Cloning Kit	Takara Bio	121416
Phusion High-Fidelity DNA Polymerase	New England BioLabs	M0530S
Deposited Data		
Sequencing data for mRNA half-life analysis	This paper	GEO: GSE114560
Pab1 RIP-Seq	Costello et al., 2015	13059_2014_559_MOESM2_ESM.xlsx
Poly(A) tail lengths	Subtelny et al., 2014	GSE52809_Cerevisiae_total.txt
Experimental Models: Organisms/Strains		
yJC244	Nonet et al., 1987	MATa, *ura3-52*, *his3-200, leu2-3.112*, *rpb1-1*
yJC1257	This study	MATa, *ura3-52*, *his3-200*, *leu2-3.112*, *rpb1-1*, *caf1::HIS3*

(*Continued on next page*)

Continued

REAGENT or RESOURCE	SOURCE	IDENTIFIER
yJC1347	This study	MATa, *ura3-52*, *his3-200*, *leu2-3.112*, *rpb1-1*, *ccr4::NEO*
yJC1892	Presnyak et al., 2015	MATa, ura3, leu2, his3, met15 [pGAL-OPT-pG, URA3]
yJC1893	Presnyak et al., 2015	MATa, ura3, leu2, his3, met15 [pGAL-NON-OPT-pG, URA3]
yJC1961	Presnyak et al., 2015	MATa, ura3, his3, leu2, met15, ccr4:NEO [pGAL-OPT-pG, URA3]
yJC1962	Presnyak et al., 2015	MATa, ura3, leu2, his3, met15, ccr4::NEO [pGAL-NON-OPT-pG, URA3]
yJC1990	This study	MATa, ura3, leu2, his3, met15, dhh1::NEO [pGAL-OPT-pG, URA3]
yJC1991	This study	MATa, ura3, leu2, his3, met15, dhh1::NEO [pGAL-NON-OPT-pG, URA3]
yJC2318	This study	MATa, ura3-52, his3-200, leu2-3.112, rpb1-1, ccr4::NEO [pGAL-OPT-pG, URA3]
yJC2319	This study	MATa, ura3-52, his3-200, leu2-3.112, rpb1-1, ccr4::NEO [pGAL-NON-OPT-pG, URA3]
yJC2320	This study	MATa, ura3-52, his3-200, leu2-3.112, rpb1-1 [pGAL-OPT-pG, URA3]
yJC2321	This study	MATa, ura3-52, his3-200, leu2-3.112, rpb1-1 [pGAL-NON-OPT-pG, URA3]
yJC2324	This study	MATa, ura3-52, his3-200, leu2-3.112, rpb1-1, caf1::HIS3 [pGAL-OPT-pG, URA3]
yJC2325	This study	MATa, ura3-52, his3-200, leu2-3.112, rpb1-1, caf1::HIS3 [pGAL-NON-OPT-pG, URA3]
yJC2364	This study	MATa, ura3, his3, leu2, met15, caf1::NEO [pGAL-OPT-pG, URA3]
yJC2365	This study	MATa, ura3, his3, leu2, met15, caf1::NEO [pGAL-NON-OPT-pG, URA3]
yJC2499	Radhakrishnan et al., 2016	MATa, ura3, leu2, his3, met15 [pGAL-optimal FLAG-0% HIS, URA3]
yJC2504	Radhakrishnan et al., 2016	MATa, ura3, leu2, his3, met15 [pGAL-optimal FLAG-50% HIS, URA3]
yJC2509	Radhakrishnan et al., 2016	MATa, ura3, leu2, his3, met15 [pGAL-optimal FLAG-100% HIS, URA3]
yJC2591	This study	MATa, ura3, leu2, his3, met15 [pGAL-SL-OPT-pG, URA3]
yJC2592	This study	MATa, ura3, leu2, his3, met15 [pGAL-SL-NON-OPT-pG, URA3]
yJC2658	This study	MATa, ura3, his3, leu2, met15, ccr4::NEO [pGAL-SL-OPT-pG, URA3]
yJC2659	This study	MATa, ura3, his3, leu2, met15, ccr4::NEO [pGAL-SL-NON-OPT-pG, URA3]
yJC2660	This study	MATa, ura3, his3, leu2, met15, caf1::NEO [pGAL-SL-OPT-pG, URA3]
yJC2661	This study	MATa, ura3, his3, leu2, met15, caf1::NEO [pGAL-SL-NON-OPT-pG, URA3]
yJC2666	This study	MATa, ura3, leu2, his3, met15, dhh1::NEO [pGAL-SL-OPT-pG, URA3]
yJC2667	This study	MATa, ura3, leu2, his3, met15, dhh1::NEO [pGAL-SL-NON-OPT-pG, URA3]
yJC2709	This study	MATa, ura3, his3, leu2, met15, caf1::NEO [pGAL-optimal FLAG-0% HIS, URA3]
yJC2710	This study	MATa, ura3, his3, leu2, met15, caf1::NEO [pGAL-optimal FLAG-50% HIS, URA3]

(*Continued on next page*)

Continued		
REAGENT or RESOURCE	SOURCE	IDENTIFIER
yJC2711	This study	MATa, ura3, his3, leu2, met15, caf1::NEO [pGAL-optimal FLAG-100% HIS, URA3]
yJC2724	This study	MATa, ura3, his3, leu2, met15, ccr4::NEO [pGAL-optimal FLAG-0% HIS, URA3]
yJC2725	This study	MATa, ura3, his3, leu2, met15, ccr4::NEO [pGAL-optimal FLAG-50% HIS, URA3]
yJC2726	This study	MATa, ura3, his3, leu2, met15, ccr4::NEO [pGAL-optimal FLAG-100% HIS, URA3]
Oligonucleotides		
DNA and RNA sequences	This paper	See Table S2
Recombinant DNA		
pJC134	This study	PGK1pG with stem loop at 5′ UTR (under control of GAL1 UAS)
pJC672	Presnyak et al., 2015	PGK1pG reporter with OPT ORF (under control of GAL1 UAS)
pJC673	Presnyak et al., 2015	PGK1pG reporter with NON-OPT ORF (under control of GAL1 UAS)
pJC857	Radhakrishnan et al., 2016	0% optimal HIS3 with N-terminal FLAG tag (GAL1 promoter)
pJC862	Radhakrishnan et al., 2016	50% optimal HIS3 with N-terminal FLAG tag (GAL1 promoter)
pJC867	Radhakrishnan et al., 2016	100% optimal HIS3 with N-terminal FLAG tag (GAL1 promoter)
pJC929	This study	pJC134 with SpeI before start codon and XhoI sites after stop codon of PGK1 ORF
pJC930	This study	PGK1pG reporter with stem loop at 5′ of OPT ORF (under control of GAL1 UAS)
pJC931	This study	PGK1pG reporter with stem loop at 5′ of NON-OPT ORF (under control of GAL1 UAS)
LP_P24-1	Stowell et al., 2016	MultiBac expression vector for *S. pombe* Ccr4-Not
LP_P24-2	Stowell et al., 2016	MultiBac expression vector for *S. pombe* Ccr4-Not (Ccr4 E387A)
LP_P24-3	Stowell et al., 2016	MultiBac expression vector for *S. pombe* Ccr4-Not (Caf1 D53A)
LP_P24-4	Stowell et al., 2016	MultiBac expression vector for *S. pombe* Ccr4-Not (Ccr4 E387A, Caf1 D53A)
LP_P22-9	This study	pGEX6P-2 expression vector for *S. pombe* Ccr4(EEP) (res 331-621)
LP_P24-5	This study	pET28a expression vector for *S. pombe* Caf1(FL) (res 1-335)
LP_P22-10	This study	pGEX6P-2 expression vector for *S. pombe* Pab1(FL) (res1-653)
LP_P22-11	This study	pGEX6P-2 expression vector for *S. pombe* Pab1 (res80-653)
LP_P22-12	This study	pGEX6P-2 expression vector for *S. pombe* Pab1ΔC (res80-576)
LP_P22-13	This study	pGEX6P-2 expression vector for *S. pombe* Pab1ΔPC (res80-441)
LP_P22-14	This study	pGEX6P-2 expression vector for *S. pombe* Pab1(RRM1mut) (res80-653; Y83A)
LP_P22-15	This study	pGEX6P-2 expression vector for *S. pombe* Pab1(RRM2mut) (res80-653; F171A)
LP_P22-16	This study	pGEX6P-2 expression vector for *S. pombe* Pab1(RRM3mut) (res80-653; Y264A)
LP_P22-17	This study	pGEX6P-2 expression vector for *S. pombe* Pab1(RRM4mut) (res80-653; F367A)

(*Continued on next page*)

Continued		
REAGENT or RESOURCE	SOURCE	IDENTIFIER
Software and Algorithms		
ImageJ	NIH	https://imagej.nih.gov/ij/
GraphPad Prism 6	GraphPad	https://www.graphpad.com/scientific-software/prism/
switchANALYSIS	Dynamic Biosensors	https://www.dynamic-biosensors.com/software/
ImageQuant	GE Healthcare	TL 5.2
Bowtie	Langmead et al., 2009	http://bowtie-bio.sourceforge.net
Samtools	Li et al., 2009	http://samtools.sourceforge.net/
Cufflinks	Trapnell et al., 2010	http://cole-trapnell-lab.github.io/cufflinks/cuffdiff/
R v.3.3.2	The R Foundation for Statistical Computing	https://www.r-project.org/
RStudio Desktop v.1.1.383	RStudio	https://www.rstudio.com/products/rstudio/

CONTACT FOR REAGENT AND RESOURCE SHARING

Further information and requests for resources should be directed to and will be fulfilled by the Lead Contact, Lori Passmore (passmore@mrc-lmb.cam.ac.uk).

EXPERIMENTAL MODEL AND SUBJECT DETAILS

Recombinant proteins Pab1, Ccr4 and Caf1 were expressed in *Escherichia coli* BL21 Star (DE3) cells grown in 2 × TY media. Recombinant Ccr4-Not and Caf1-Ccr4 were expressed in the *Spodoptera frugiperda Sf9* cell line. *Saccharomyces cerevisiae* strains used in this study are listed in the Key Resources Table. All yeast strains were grown at 24°C in synthetic media supplemented with the appropriate amino acids and either 2% glucose, 2% raffinose/1% sucrose or 2% galactose/1% sucrose. Yeast was harvested at mid-log phase (OD_{600nm} = 0.36–0.55).

METHOD DETAILS

Protein Purification

Intact Ccr4-Not complex was purified after overexpression of the seven core subunits of the *Schizosaccharomyces pombe* complex (Ccr4, Caf1, Not1, Not2, Not3, Not4/Mot2 and Rcd1/Caf40) in *Sf*9 cells (Stowell et al., 2016).

The Caf1-Ccr4 heterodimeric complex was prepared from the *Sf*9 lysate used for Ccr4-Not expression as these subunits were expressed in molar excess and were captured using the Strep II tag on the Caf1 subunit. This sample was separated from Ccr4-Not with a 5 mL HiTrap Q HP column (GE Healthcare) equilibrated in buffer A (20 mM HEPES pH 7.4, 50 mM NaCl, 0.1 mM TCEP) eluted over a 10-column volume gradient into buffer A with 1 M NaCl. The pooled eluate was applied to a Superdex 200 10/300 GL size-exclusion column (GE Healthcare) equilibrated in buffer A. Peak fractions were concentrated with an Amicon Ultra 50 kDa MWCO centrifugal concentrator (Millipore) and stored at −80°C.

For preparation of isolated nucleases, DNA encoding *S. pombe CAF1* and *CCR4* were synthesized with codon optimization (Genscript). Full-length *CAF1* was cloned into a modified pET28a plasmid for expression as an N-terminal hexahistidine fusion in BL21 Star (DE3) *E. coli* (Thermo Fisher Scientific). The sequence encoding the Ccr4 EEP domain (amino acids 331–621) was amplified using primers Ccr4_Nuc_Fwd and Ccr4_Nuc_Rev (Table S2). This was cloned into pGEX-6P-2 plasmid using an In-Fusion HD Cloning Kit (Clontech) for overexpression as an N-terminal GST-fusion in *E. coli* BL21 Star (DE3) cells. Transformed cells were grown at 37°C to an $A_{600\ nm}$ of 0.6 before the temperature was reduced to 18°C and protein expression induced by the addition of IPTG to 1 mM (Caf1) or 0.5 mM (Ccr4 EEP). Growth was continued for 18 hr before cells were harvested by centrifugation and flash frozen for storage at −80°C.

Caf1-expressing cells were defrosted and lysed by sonication in lysis buffer (50 mM HEPES pH 8, 500 mM NaCl, 2 mM $MgCl_2$, 1 mM TCEP) supplemented with protease inhibitor cocktail (Roche). Caf1 was purified from the lysate with Ni-NTA affinity resin (QIAGEN). The resin was washed with lysis buffer supplemented with 20 mM imidazole, and the protein eluted in buffer B (20 mM HEPES pH 8, 300 mM NaCl, 2 mM $MgCl_2$, 0.5 mM TCEP and 250 mM imidazole). The hexahistidine tag was cleaved by treatment with 3C protease. The sample was diluted to 150 mM NaCl before application to a 5 mL HiTrap Q HP column and elution over a 10-column volume gradient into buffer B with 1 M NaCl. Peak fractions were pooled and applied to a HiLoad Superdex 75 26/60 pg column equilibrated in 20 mM HEPES pH 8, 150 mM NaCl, 2 mM $MgCl_2$, 0.5 mM TCEP. The protein was concentrated with an Amicon Ultra 10 kDa MWCO concentrator (Millipore) and stored at −80°C.

Ccr4 EEP-expressing cells were sonicated in lysis buffer containing 50 mM Tris-HCl pH 8, 250 mM KCl, 1 mM TCEP, and protein was purified from the lysate with Glutathione Sepharose 4B (GE Healthcare). The resin was washed with buffer A containing 1 M NaCl, and protein was eluted in buffer A supplemented with 50 mM glutathione. Nucleic acid contaminants were removed by application of the sample to a 5 mL HiTrap Q HP column equilibrated in buffer A and eluted over a 10-column volume gradient into buffer A with 1 M NaCl. The sample was treated with 3C protease to cleave off the GST tag (16 hr at 4°C), and then applied to a 5 mL HiTrap Q HP column run in the conditions described above. The sample was then applied to a 5 mL HiTrap Heparin HP column equilibrated in buffer A and eluted over a 10-column volume gradient into buffer A with 1 M NaCl. Peak fractions were pooled and applied to a Superdex 200 10/300 GL size-exclusion column (GE Healthcare) equilibrated in buffer A. Pure protein was concentrated with an Amicon Ultra 10 kDa MWCO concentrator and stored at −80°C.

DNA encoding Pab1 amino acids 80–653 was amplified from *S. pombe* cDNA using primers Pab1_res80_Fwd and Pab1_Rev (Table S2) and cloned into pGEX-6P-2 plasmid using an In-Fusion HD Cloning Kit (Clontech). DNA encoding variants Pab1ΔC and Pab1ΔPC were amplified from this vector with primers Pab1_res80_Fwd and Pab1ΔC_Rev or Pab1ΔPC_Rev. These were also cloned into pGEX-6P-2 plasmid. Pab1 was expressed and purified as described above for Ccr4 (EEP nuclease domain).

PCR-based site-directed mutagenesis was performed with a Quikchange Lightning Multi Mutagenesis Kit (Agilent) to generate mutations in Pab1 (RRM1mut: Y83A, RRM2mut: F171A, RRM3mut: Y264A, RRM4mut: F367A). A single primer was used to introduce each modification: Pab1_Mut1_QC, Pab1_Mut2_QC, Pab1_Mut3_QC, and Pab1_Mut4_QC (Table S2). Pab1 variants were purified as described for wild-type versions.

Deadenylation Assays

Deadenylation activity was measured (Webster et al., 2017) in 20 mM PIPES pH 6.8, 10 mM KCl, 45 mM NaCl, 2 mM $Mg(OAc)_2$, 0.1 mM TCEP (includes components added with protein factors) at 22°C. Ccr4-Not and Caf1-Ccr4 were prepared at 1 μM (10×) in 20 mM HEPES pH 7.4, 400 mM NaCl, 2 mM $Mg(OAc)_2$, 0.1 mM TCEP and added to a final concentration of 100 nM in the reaction. Caf1 and Ccr4 (EEP domain) were prepared in the same buffer and added to final concentrations of 5 μM and 1 μM respectively. Pab1 was prepared in 20 mM HEPES pH 7.4, 50 mM NaCl, 0.1 mM TCEP and added to a final concentration of 200 nM or 400 nM in the reaction (for 1 or 2 Pab1 molecules per RNA respectively). Pab1 was incubated with RNA for 10 min at 22°C prior to the addition of enzyme to allow protein-RNA binding to reach equilibrium.

23-mer-A30, A30 and 20-mer-A10 RNAs (Table S2) were synthesized with a 5′ 6-FAM fluorophore label (Integrated DNA Technologies). The 20-mer-A60 RNA was generated by *in vitro* transcription: A modified pUC57 vector containing a T7 promoter and the encoded RNA sequence was linearized with BsaI restriction enzyme to generate the DNA template (Webster et al., 2017). *In vitro* transcription was performed using standard procedures (Wolf et al., 2014).

200 nM RNA was used in each reaction. Reactions were stopped at the indicated time points by addition of 2 × denaturing loading dye (95% formamide, 10 mM EDTA, 0.01% w/v bromophenol blue). Samples were applied to TBE (Tris-borate-EDTA)-polyacrylamide gels containing 7 M urea (20% acrylamide for 23-mer-A30, 20-mer-A10 and A30; 14% acrylamide for 20-mer-A60) and run at 400 V in 1 × TBE running buffer. Gels were scanned with a Typhoon FLA-7000 directly for 5′ 6-FAM labeled RNA (20-mer-A10, 23-mer-A30 and A30) or following staining of the gel with SYBR Green II for unlabeled RNA (20-mer-A60). Densitometric analysis was performed with ImageJ (Schneider et al., 2012; Webster et al., 2017). Poly(A) tail lengths were calibrated using RNA markers with no tail and tails of known length. Intermediate tail lengths were calculated by counting bands on gels with single-nucleotide resolution (Webster et al., 2017). Average rates of deadenylation were calculated by linear regression of modal tail length plots. All results are representative of experiments performed in triplicate. The greatest source of error was determined to be in applying a linear fit and therefore we calculated the uncertainty in the average rate as the 95% confidence interval of the slope.

For the analysis of Pab1 dissociation during deadenylation, reactions were stopped by the addition of 2 × non-denaturing stop solution (20 mM Tris-HCl pH 8, 5 mM EDTA, 10% v/v glycerol, 0.1% Orange G). Samples were applied to 6% TBE-polyacrylamide non-denaturing gels and electrophoresis was performed at 100 V in 1 × TBE running buffer. Gels were scanned with a Typhoon FLA-7000.

Electrophoretic Mobility Shift Assays

Binding reactions (10 μl) were prepared by adding Pab1 at the indicated molar excess (0.5 × = 100 nM, 1 × = 200 nM, 2 × = 400 nM, 3 × = 600 nM, 4 × = 800 nM) to RNA (200 nM) in 20 mM PIPES pH 6.8, 10 mM KCl, 90 mM NaCl, 2 mM $Mg(OAc)_2$, 0.1 mM TCEP. The sample was incubated for 15 min at 22°C before the addition of 6 × loading dye (30% glycerol and 0.2% w/v orange G). Samples were applied to 6% TBE-polyacrylamide non-denaturing gels and electrophoresis was performed at 100 V in 1 × TBE running buffer. Gels were scanned with a Typhoon FLA-7000 directly for 5′ 6-FAM labeled RNA (20-mer-A10, 23-mer-A30 and A30) or following staining of the gel with SYBR Green II for unlabeled RNA (20-mer-A60).

Fluorescence Polarization Assays

A two-fold protein dilution series was prepared in 20 mM HEPES pH 7.4, 150 mM NaCl. Proteins were incubated for 2 hr at 22°C with 0.2 nM 5′ 6-FAM RNA (synthesized by IDT, Table S2). Fluorescence polarization was measured with a PHERAstar Plus microplate reader (BMG Labtech). Dissociation constants were estimated by non-linear regression with a one-site binding curve in *GraphPad Prism 6*. Error bars indicate the standard deviation in five replicate measurements.

SwitchSENSE Kinetic Analysis

Kinetic measurements were performed using a DRX series instrument with a MPC-48-2-Y1 chip (Dynamic Biosensors). Hybrid oligonucleotides were synthesized (IDT) with the RNA of interest (A30, or N20A_n, Table S2) at the 5′ end followed by single-stranded DNA complementary in sequence to the fluorescently labeled oligonucleotide on the chip. Annealing was performed by flowing 500 nM oligonucleotide over the chip for 4 min in a buffer of 20 mM HEPES pH 7.4, 40 mM NaCl and 0.001% Tween-20. Analysis of Pab1 kinetics was performed by application of 25 nM Pab1 in a buffer of 20 mM HEPES pH 7.4, 150 mM NaCl and 0.001% Tween-20. Binding experiments were performed at 20°C with a flow rate of 30 μl/min. The dynamic response represents the change in nanolever switch speed on the timescale 0–4 μsec (Langer et al., 2013). Data points from dissociation experiments 20-mer-A10, 20-mer-A15 and A30 were averaged in 10 s intervals to improve the signal-to-noise. Kinetic constants were estimated by fitting of an exponential function with GraphPad Prism 6.

Yeast Strains and Growth Conditions

Yeast strains used in this study are listed in the Key Resources Table. All strains were grown at 24°C in synthetic media supplemented with the appropriate amino acids and either 2% glucose, 2% raffinose/1% sucrose or 2% galactose/1% sucrose. Cells were harvested at mid-log phase (OD_{600nm} = 0.36–0.55).

RNA Labeling and RNase A/T1 Digestion

Yeast (*rpb1-1*, *rpb1-1 ccr4*Δ, and *rpb1-1 caf1*Δ) were grown to mid-log phase in minimal synthetic media (pH 6.5). At mid-log phase, transcription was repressed by shifting cells to 37°C, and cell aliquots were harvested at 0, 3, 6, 8, 10, 15, 20, 30, 45, 60 min. Total RNA was isolated from each sample as described previously (Geisler et al., 2012).

[5′ ^{32}P] cytidine 3′ 5′ bisphosphate (pCp) was prepared by combining 1 μL 833 μM 3′ CMP, 33 μL 25 μM [γ^{32}P] ATP (Perkin Elmer NEG035C), 4 μL 10 × OptiKinase buffer, and 2 μL OptiKinase (USB 78334Y) and incubating for 1 hr at 37°C before heat inactivating at 65°C for 10 min. 2 μg of each total RNA sample isolated above was then [5′ ^{32}P] pCp labeled at 4°C overnight after combining the RNA with 2 μL [5′ ^{32}P] pCp in a 10 μL reaction containing 1 × T4 RNA ligase buffer, 10% DMSO, 0.5 mM rATP, and 10 units T4 RNA ligase (Thermo Fisher Scientific EL0021). Samples were purified by passing through two 1 mL G50 Sephadex columns after adding 90 μL of HS buffer (20 mM Tris-HCl pH 7.5, 10 mM EDTA and 300 mM NaCl).

Next, 25 μL of each pCp-labeled RNA sample was RNase A and RNase T_1 digested at 30°C for 30 min in 1 × RNase A/T_1 digestion buffer [20 mM Tris-HCl pH 7.5, 1 mM $MgCl_2$, 100 mM KCl, and 1 mg/ml tRNA (Sigma R4251)] using 1 unit RNase T_1 (Sigma R1003) and 10 μg RNase A (Sigma R6513) in a 100 μL reaction. The samples were extracted with phenol/chloroform buffered with LET (25 mM Tris-HCl, pH 8.0, 100 mM LiCl, 20 mM EDTA, pH 8.0) and then ethanol precipitated with ammonium acetate in the presence of 1 μL GlycoBlue (Thermo Fisher Scientific AM9515). The precipitated RNA was run on denaturing 12% polyacrylamide sequencing gels and dried before exposing to a phosphorimager screen.

Reporter Construction

The plasmids and oligonucleotides used in this study are listed in the Key Resources Table and Table S2. To construct the synthetic reporters containing the stem-loop at the 5′ UTR of OPT (pJC672) and NON-OPT (pJC673) reporters, SpeI and XhoI sites were introduced directly before the start codon and after the stop codon of the *PGK1* ORF in pJC134 (SL-PGK1pG) by oJC3208/3209 and oJC2379/2380, respectively to create pJC929. The OPT and NON-OPT ORFs were subsequently cloned into this plasmid by using SpeI and XhoI sites to generate pJC930 (SL-OPT) and pJC931 (SL-NON-OPT), respectively.

Transcriptional Shut-Off and Pulse-Chase

For the *GAL1* promoter shut-off experiment, cells were grown at 24°C in synthetic media with 2% galactose/1% sucrose to allow for expression of the reporters. Cells were shifted to synthetic media without sugar at an OD_{600nm} = 0.4, and then transcription was repressed by adding glucose to a final concentration of 4%. Aliquots were collected at the time points indicated in the figures.

For the *GAL1* promoter pulse-chase experiments, cells were inoculated in synthetic media containing 2% raffinose/1% sucrose to keep the *GAL1* promoter off. Once cells reached to OD_{600nm} = 0.36, they were shifted to synthetic media without sugar and the transcription of *GAL1* promoter was activated by adding 2% galactose for 8 min. After an 8-min induction of transcription, for WT and *dhh1*Δ strains (Figure 5B), cells were shifted to synthetic media without sugar and then transcription was repressed by adding glucose to a final concentration of 4%; for *rpb1-1*, *rpb1-1/caf1*Δ and *rpb1-1/ccr4*Δ strains (Figure 6B), transcription was repressed by adding glucose to a final concentration of 4% and shifting cells to 37°C. Cells were collected after transcriptional inhibition at the time points indicated in the figures.

Total RNA was extracted by phenol/chloroform/LET (25 mM Tris, pH 8.0, 100 mM LiCl, 20 mM EDTA) and precipitated by 95% EtOH. 30-40 μg of RNA was separated on 1.4% agarose-formaldehyde gels at 100 V for 1.5 hr or 6% high resolution polyacrylamide gels at 400 V for 14.5 hr, transferred to nylon membranes and probed with ^{32}P-labeled antisense oligonucleotides complementary to poly(G) (oJC168), *HIS3* (oJC2564), and *SCR1* (oJC306). Blots were exposed to PhosphorImager screens, scanned by Typhon 9400, and quantified by ImageQuant software to determine half-lives. Quantification of mRNA half-life (Figures 6A and 6C) was performed

following normalization to *SCR1* RNA, which is not shown. The deadenylation rates of OPT and NON-OPT mRNAs (Figures 5B and 6B) were determined by calculating changes of shortest poly(A) tail lengths in time points which have more than A10 on blots in Figures 5B and S7.

Global mRNA Half-Life Analysis

RNA-seq experiment and half-lives analysis were performed as described in Presnyak et al. (2015). Briefly, *rpb1-1* and *rpb1-1/caf1*Δ cells were grown to mid-log phase at 24°C and shifted to a non-permissive temperature (37°C) to inactivate RNA polymerase II. Cells were collected at various time points after the inhibition of transcription. RNA was extracted and 1 ng of ERCC Phage NIST spike-ins was added. Libraries were prepared by using Illumina TruSeq Stranded Total RNA and mRNA library prep kits, quantified by an Agilent Bioanalyzer and sequenced by using paired-end 100 bp reads with an index read on Illumina HiSeq2000. Sequencing data are available at Gene Expression Omnibus (https://www.ncbi.nlm.nih.gov/geo) with accession number GSE114560.

Reads were aligned to the SacCer2 *S. cerevisiae* reference genome by Bowtie v0.12.7 (Langmead et al., 2009) and the aligned reads were further converted into bam format and indexed by Samtools v0.1.18 (Li et al., 2009). Gene FPKM values were calculated by Cufflinks v1.3.0 (Trapnell et al., 2010) and annotated to the SacCer2 SGD gene annotation downloaded from the UCSC browser. The raw FPKM values were normalized to the spike-ins reads.

The expression levels of each gene at each time point were normalized to the initial expression level (0-min time point). The half-life for each gene was determined by fitting data into an exponential decay curve. Dubious and unverified ORFs, genes for which the average absolute residual was greater than 0.14, and genes with an estimated half-life longer than the measured time course were excluded. Each transcript's average optimality was calculated using the definitions of codon optimality in Pechmann and Frydman (2013), then boxplots of mRNA half-lives in *caf1*Δ cells relative to wild-type cells for each optimality bin were generated using ggplot2 / geom_boxplot in R. Base R functions were used to perform ANOVA ($p = 1.05 \times 10^{-14}$), then Tukey's Honest Significant Difference test for pairwise comparisons with the < 40% optimality bin (Figure 6D).

Analysis of Pab1 Occupancy

Pab1 binding per nucleotide of poly(A) tail for each *S. cerevisiae* transcript was calculated as follows. First, we converted "logFC" values in "13059_2014_559_MOESM2_ESM.xlsx" (Costello et al., 2015) to fold-change, then divided these values by mean poly(A) tail lengths derived from "GSE52809_Cerevisiae_total.txt" (Subtelny et al., 2014). Next, each transcript's average optimality was calculated using the definitions of codon optimality in Pechmann and Frydman (2013), then boxplots of Pab1 binding per nucleotide of poly(A) tail for each optimality bin were generated using ggplot2 / geom_boxplot in R. Base R functions were used to perform ANOVA ($p < 2 \times 10^{-16}$), then Tukey's Honest Significant Difference test for pairwise comparisons with the < 40% optimality bin (Figure 5A).

QUANTIFICATION AND STATISTICAL ANALYSIS

Statistical parameters are reported in the Figures and Figure Legends.

In Vitro Deadenylation Analysis

All results of *in vitro* deadenylation analysis are representative of experiments performed in triplicate. Average rates of deadenylation were calculated by linear regression of modal tail length plots. Uncertainty is presented as the 95% confidence interval of the linear fit slope as this is the largest source of error.

Fluorescence Polarization Assays

Quantification of interaction affinity was determined by non-linear regression with a one-site binding curve. Error bars indicate the standard deviation in replicate measurements and K_D measurements are presented as the mean ± standard error.

SwitchSENSE Kinetic Analysis

Analysis of Pab1 dissociation rate was performed in triplicate for each RNA sequence and representative sensograms are shown. Rate constants and half-lives for dissociation are presented as the mean ± standard error. Analysis of Pab1 association rate was performed in triplicate for each protein concentration. Linear regression was used to determine the kinetic constant for association (k_{on}), which is presented as the mean ± standard error.

In Vivo Deadenylation Analysis

Rates of *in vivo* deadenylation were analyzed by quantifying the shortest poly(A) tail lengths at each time point. In Figure 6B, error bars indicate standard deviation in triplicate measurements.

In Vivo mRNA Half-Life Analysis

Reporter mRNA half-lives were calculated by densitometry with normalization to *SCR1* RNA control and are presented as the mean ± standard deviation. For transcriptome-wide analysis of mRNA half-lives, the expression levels of each gene at each time point were

normalized to the initial expression level (0-min time point). The half-life for each gene was determined by fitting data into an exponential decay curve. Boxplots of mRNA half-lives in *caf1*Δ cells relative to wild-type cells for each optimality bin were generated using ggplot2 / geom_boxplot in R. Base R functions were used to perform ANOVA ($p = 1.05 \times 10^{-14}$), then Tukey's Honest Significant Difference test for pairwise comparisons with the < 40% optimality bin.

Pab1 Enrichment

Boxplots of Pab1 binding per nucleotide of poly(A) tail for each optimality bin were generated using ggplot2 / geom_boxplot in R. Base R functions were used to perform ANOVA ($p < 2 \times 10^{-16}$), then Tukey's Honest Significant Difference test for pairwise comparisons with the < 40% optimality bin (Figure 5A).

DATA AND SOFTWARE AVAILABILITY

The accession number for the raw RNA sequencing data files used for the calculation of global mRNA half-lives is NCBI GEO: GSE114560.

Molecular Cell, Volume *70*

Supplemental Information

mRNA Deadenylation Is Coupled to Translation Rates by the Differential Activities of Ccr4-Not Nucleases

Michael W. Webster, Ying-Hsin Chen, James A.W. Stowell, Najwa Alhusaini, Thomas Sweet, Brenton R. Graveley, Jeff Coller, and Lori A. Passmore

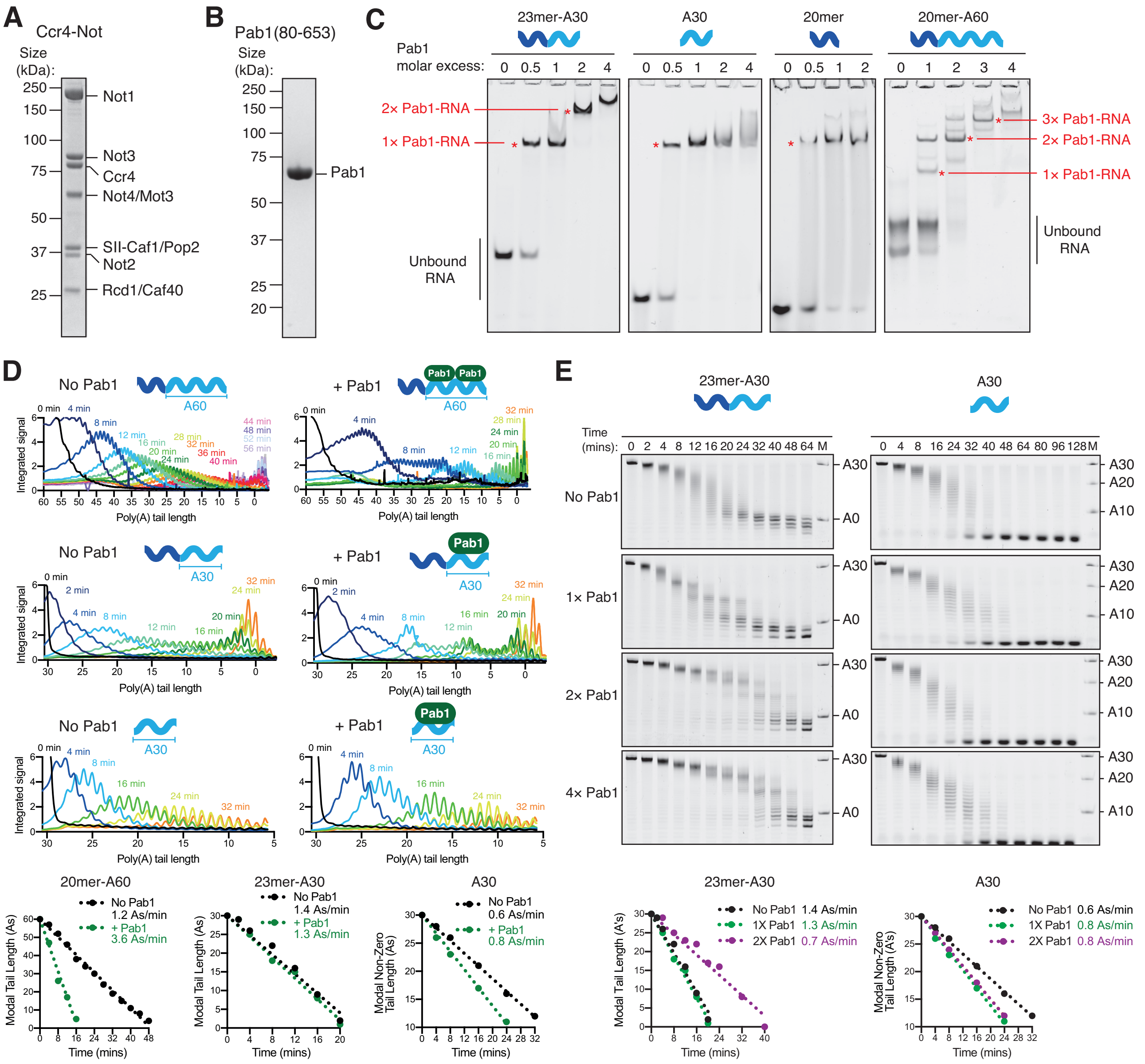

Figure S1. Reconstitution and analysis of deadenylation on Pab1-bound RNAs, Related to Figure 1.

(**A**) Coomassie-stained SDS-PAGE of *S. pombe* Ccr4-Not complex purified after overexpression in *Sf*9 insect cells. (**B**) Coomassie stained SDS-PAGE of *S. pombe* Pab1 (residues 80-653) purified following overexpression in *E. coli*. (**C**) Electrophoretic mobility shift assays (EMSAs) showing the Pab1-RNA complexes used as substrates for deadenylation assays. Purified Pab1 was mixed with RNA in the indicated molar ratio relative to RNA, and resolved on a 6% non-denaturing polyacrylamide gel. Gels containing 23mer-A30, A30, 20mer and 20mer-A60 were imaged to detect the 5′ fluorescent label. Gel containing 20mer-A60 was stained with SYBR Green II before scanning for fluorescence. Notably, Pab1 also binds to the non-poly(A) 20mer RNA. (**D**) Densitometric analyses of gels (top) and plots of the most abundant RNA poly(A) tail

length versus time (bottom) (Webster et al., 2017) for assays with 20mer-A60, 23mer-A30, and A30. Linear regression was applied to obtain the indicated reaction rates. 95% confidence intervals: ±0.04 (20mer-A60 no Pab1), ±0.6 (20mer-A60 + Pab1), ±0.1 (23mer-A30 no Pab1), ±0.1 (23mer-A30 + Pab1), ±0.02 (A30 no Pab1), ±0.05 (A30 + Pab1). (**E**) Deadenylation reactions performed with Ccr4-Not and a series of Pab1 concentrations. Pab1 was mixed with RNA in the indicated molar ratio relative to RNA to form the complexes indicated in (C). Experiments were performed with 23mer-A30 (left) and A30 (right), and poly(A) tail lengths are indicated. Reaction rates were determined as in (D). 95% confidence intervals: ±0.07 (23mer-A30 2X Pab1), ±0.05 (A30 2X Pab1). Interestingly, in our deadenylation assays using an A30 substrate lacking any upstream non-poly(A) sequence, Ccr4-Not did not proceed in step-wise manner (Figures S1D and S1E). Therefore, step-wise deadenylation likely occurs because the 3′-UTR stabilizes and positions Pab1 on RNA.

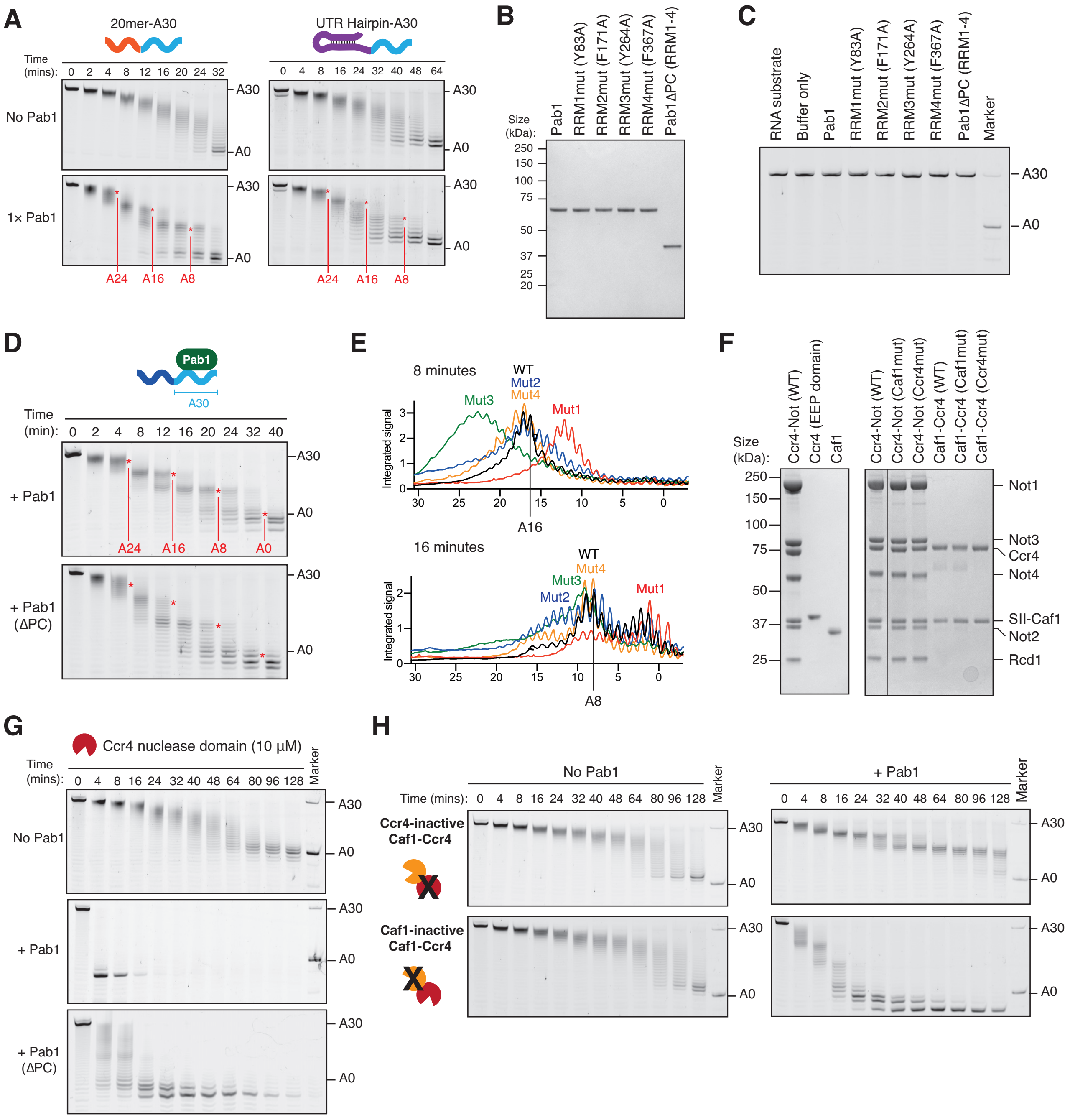

Figure S2. Pab1 differentially affects the deadenylation activities of Caf1 and Ccr4, Related to Figures 1 and 2.

(**A**) Deadenylation of a 20mer-A30 and UTR hairpin-A30 RNA by Ccr4-Not in the presence of Pab1 showing ~8 nucleotide steps. The stepwise pattern of deadenylation was also observed with these RNA substrates, indicating it is not dependent on the specific sequence of the 23mer-A30 RNA. (**B**) Coomassie-stained SDS-PAGE of purified Pab1 variant proteins. The concentration of each sample was normalized to ensure an equivalent amount of Pab1 was added to the deadenylation assays. (**C**) Control deadenylation reactions showing the absence of RNase contamination in samples of

purified Pab1 proteins. Pab1 at a concentration equivalent to that used in assays with Ccr4-Not was incubated with fluorescein-labelled 23mer-A30 RNA for 64 minutes and RNA was resolved on a denaturing polyacrylamide gel. (**D**) Deadenylation of a 30-adenosine RNA by Ccr4-Not in the presence of full-length Pab1 and a Pab1 variant containing only the RRM domains (Pab1ΔPC; residues 80–362). Asterisks indicate transient pausing of Ccr4-Not in the presence of wild-type Pab1. (**E**) Densitometric analysis of gels in Figure 1D shows altered step-wise deadenylation of 23mer-A30 RNA when mutations were introduced into the RRM domains of Pab1: RRM1 (Mut1), RRM2 (Mut2), RRM3 (Mut3), RRM4 (Mut4). Deadenylation in the presence of the RRM1 mutant is generally faster, suggesting that Pab1 is able to recruit Ccr4-Not to the RNA but RRM binding is weakened, eliminating the transient stalling of the nucleases when they encounter an RRM binding site. In contrast, mutation in RRM3 caused a slowing of deadenylation. (**F**) Coomassie-stained SDS-PAGE of purified Ccr4-Not subcomplexes and catalytic mutant variants. The concentration of enzyme in each sample was normalized to ensure an equivalent amount was added to deadenylation assays. Ccr4-Not (WT) lane reproduced from Figure S1. (**G**) Deadenylation of 23mer-A30 RNA by purified *S. pombe* Ccr4 EEP nuclease domain (10 μM) without Pab1 and in the presence of Pab1 or a truncated Pab1 variant containing only RRM domains 1-4 (Pab1ΔPC) with a 1:1 molar ratio of Pab1 to RNA. In this reaction, 10-fold more Ccr4 was added than in the reaction shown in Figure 2C, demonstrating that the enzyme is active in the absence of Pab1 but is accelerated more than 30-fold by its presence. (**H**) Deadenylation of 23mer-A30 RNA in the absence or presence of Pab1 (1:1 molar ratio to RNA) by purified *S. pombe* Caf1-Ccr4 dimeric subcomplex variants containing mutations that abolish the activity of either Caf1 or Ccr4.

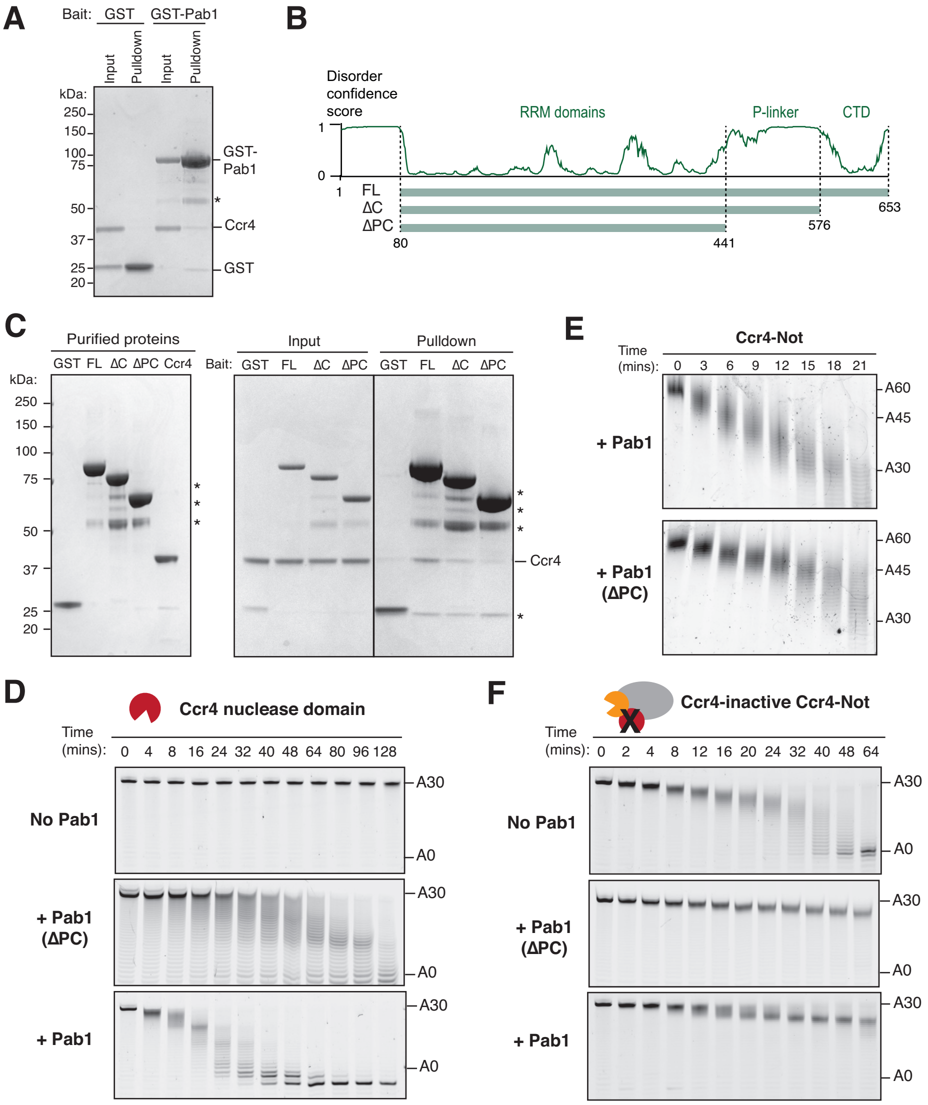

Figure S3. The C-terminal region of Pab1 interacts with Ccr4 and is important to deadenylation, Related to Figure 2.

(**A**) Pulldown assay showing the interaction between Ccr4 and immobilized GST-Pab1 is not mediated by nucleic acids. GST-Pab1 and Ccr4 were incubated with 500 U of benzonase for 30 minutes at room temperature before the experiment was performed as in Figure 2D. (**B**) Diagram showing the design of Pab1 variants based on predicted structural features of Pab1. Disorder confidence plot was generated with *DISOPRED3* (Jones and Cozzetto, 2015) and the amino acid regions of each construct are indicated. (**C**) Coomassie-stained SDS-PAGE of pull-down assays showing binding of purified Ccr4 to immobilized GST-Pab1 variants. Contaminant proteins are indicated with asterisks. (**D**) Deadenylation of 23mer-A30 RNA by Ccr4 (EEP nuclease domain; 1 μM) without Pab1, or in the presence of Pab1(ΔPC) or Pab1. Pab1-bound substrate was prepared with one Pab1 molecule per RNA. Deadenylation by isolated Ccr4 was stimulated less by a Pab1 variant lacking these domains (Pab1ΔPC) than by full-length Pab1 (Pab1: 0.94 nt/min/μmol; Pab1ΔPC: 0.23 nt/min/μmol; no Pab1: 0.04 nt/min/μmol;

also see Figure 2C). The presence of Pab1ΔPC does still increase the rate of reaction relative to when no Pab1 was added. This could be through direct interactions with Ccr4-Not and may involve allosteric effects, but a major interaction site on Pab1 is in its C-terminal region. The proline-rich linker of Pab1 had previously been shown to be important to deadenylation and mRNA stability *in vivo*. In *S. cerevisiae*, removal of this domain reduced deadenylation rates by 60–80% (Yao et al., 2007) and increased mRNA half-lives by approximately 2-fold (Simón and Séraphin, 2007). Because Pab1 self-association relies on the P-linker domain, self-association was proposed to be important to deadenylation. In our assays, the P-linker is important even in conditions when there is only one Pab1 molecule per RNA. Thus, our findings suggest that recruitment of Ccr4-Not is another critical role for the Pab1 P-linker domain. (**E**) Deadenylation of Pab1-bound A60 RNA by intact Ccr4-Not (50 nM) was impaired by removal of the C-terminal portion of Pab1 (Pab1(ΔPC)). Pab1-bound substrate was prepared with two Pab1 molecules per RNA. (**F**) Deadenylation of 23mer-A30 RNA by Ccr4-inactive Ccr4-Not (100 nM) without Pab1, or in the presence of Pab1(ΔPC) or Pab1. This shows that Pab1 RRM domains alone promote deadenylation and account for the dependence on Ccr4.

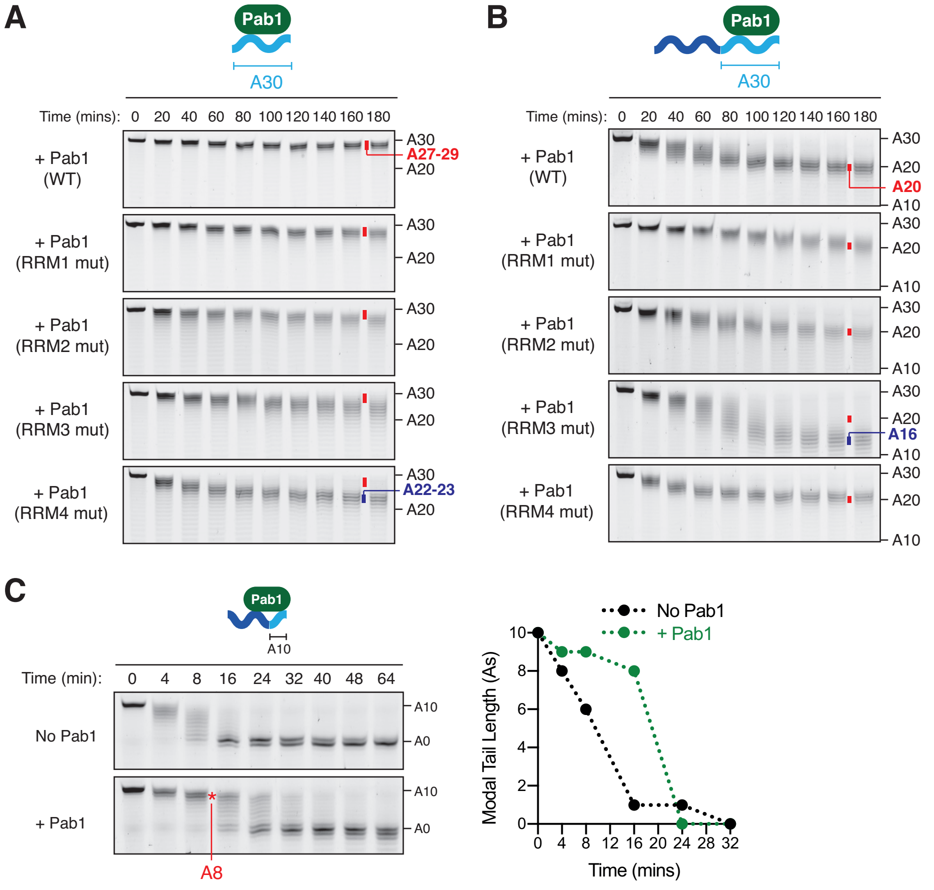

Figure S4. Mapping the position of Pab1 binding on RNA, Related to Figures 3 and 4.

(**A**) Deadenylation of a 30-adenosine RNA (without an upstream sequence) by Ccr4-inactive Ccr4-Not in the presence of Pab1 variants (1:1 molar ratio to RNA). Red markers indicate the position of the ~A28 fragment protected in the presence of wild-type Pab1. Blue marker indicates the smaller ~A22 protected fragment generated in the presence of RRM4 mutant Pab1. (**B**) Deadenylation of a 30-adenosine RNA with an upstream non-poly(A) (3′-UTR) sequence (23mer-A30) by Ccr4-inactive Ccr4-Not in the presence of Pab1 variants (1:1 molar ratio to RNA). Red markers indicate the expected position of the ~A20 protected fragment generated in the presence of wild-type Pab1. (**C**) Deadenylation of 23mer-A10 RNA by wild-type Ccr4-Not in the absence or presence of Pab1. Quantification plot of the most abundant RNA poly(A) tail length versus time is shown (right).

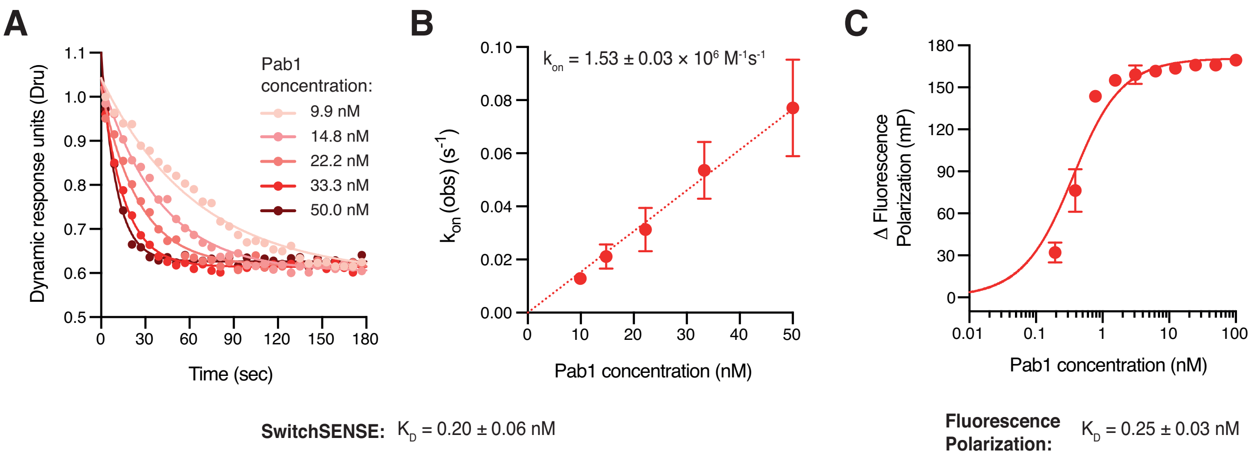

Figure S5. Kinetics of the interaction between Pab1 and polyadenosine RNA, Related to Figure 4.

(**A**) Representative switchSENSE sensograms showing the association of Pab1 with a 30-adenosine RNA at a series of protein concentrations. An exponential model was fitted to the data. (**B**) The observed association rate was determined from triplicate measurements of experiments shown in (A). Linear regression was used to determine the kinetic constant for association (k_{on}). The standard error is given. (**C**) Fluorescence polarization assay was performed with Pab1 and 5′ 6-FAM-labelled 30-adenosine RNA to validate the binding affinity (K_D) determined by switchSENSE. A one-site quadratic binding curve was fitted to the data.

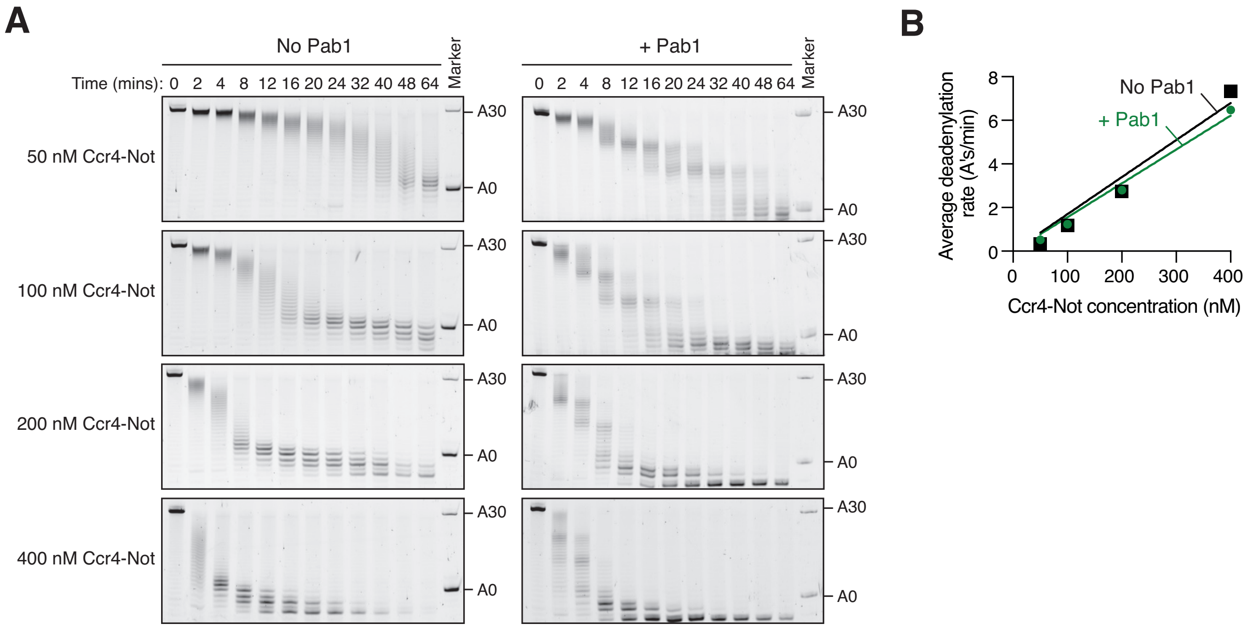

Figure S6. Pab1 does not limit the rate of Ccr4-Not-mediated deadenylation, Related to Figure 4.

(**A**) Deadenylation of 23mer-A30 RNA (200 nM) by Ccr4-Not at the indicated concentrations, in the absence or presence of Pab1 (1:1 molar ratio to RNA). (**B**) Deadenylation rates from (A) in the absence or presence of Pab1 (1:1 molar ratio to RNA) at a series of Ccr4-Not concentrations from triplicate measurements (error bars are smaller than the data points shown).

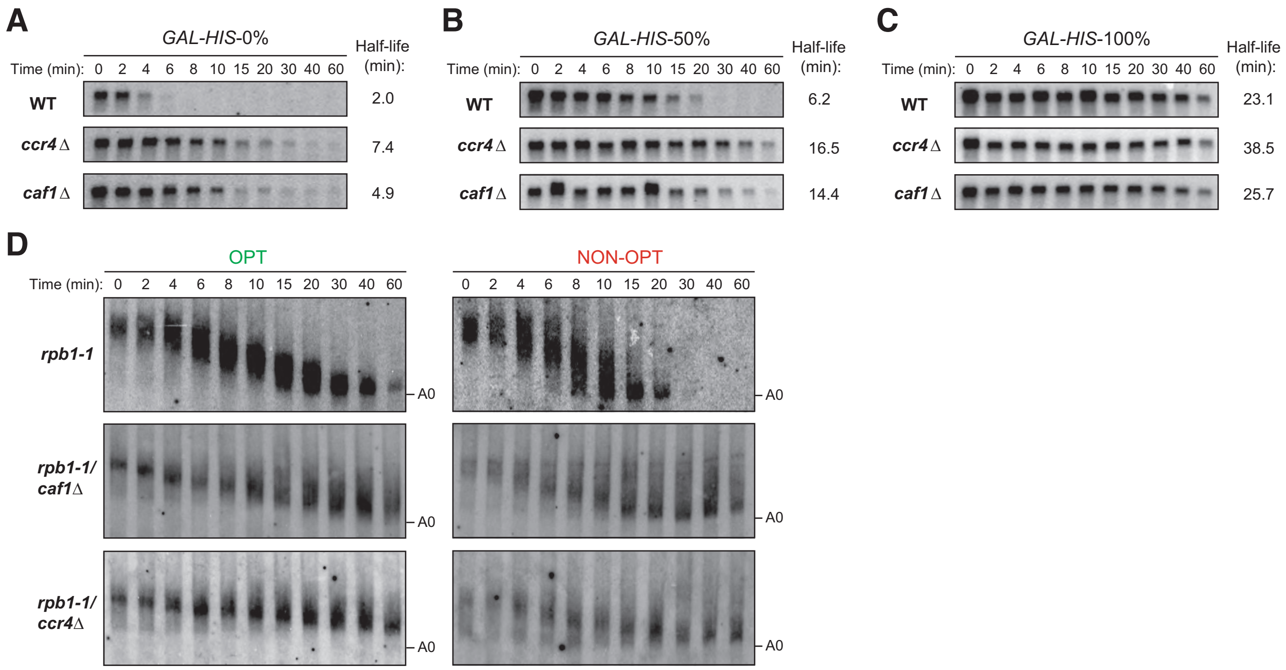

Figure S7: Caf1 preferentially destabilizes mRNAs with low codon optimality, Related to Figure 6.
(**A-C**) Northern blots of three *GAL-HIS* reporters with 0% (A), 50% (B) or 100% (C) optimality following *GAL1* transcriptional shut-off experiments in WT, *ccr4*Δ or *caf1*Δ cells. Quantification of mRNA half-life was performed following normalization to *SCR1* RNA, which is not shown. Deletion of *CAF1* preferentially stabilized mRNA with lower codon optimality (*GAL-HIS3*-0% and 50%), while deletion of *CCR4* stabilizes all three *GAL-HIS3* reporters with 0%, 50% or 100% optimality. (**D**) High-resolution polyacrylamide northern blots of the OPT and NON-OPT mRNAs following transcriptional pulse-chase experiments by inhibiting *GAL1* promoter and inactivating RNA polymerase II at 37 °C in *rpb1-1, rpb1-1/ccr4*Δ or *rpb1-1/caf1*Δ cells. A0 denotes the completely deadenylated mRNA species.

Table S1: Deadenylation rates of OPT and NON-OPT mRNAs,

Related to Figure 6.

	Deadenylation rate (nucleotides/min)*	
	OPT	NON-OPT
rpb1-1	1.70 ± 0.1	4.17 ± 0.5
*rpb1-1/caf1*Δ	1.55 ± 0.2	1.14 ± 0.1
*rpb1-1/ccr4*Δ	0.52 ± 0.1	0.88 ± 0.2

*Deadenylation rates were determined by calculating the shortest poly(A) tail length for each time point until this reaches <A10. Data are represented as the mean ± standard deviation for experiments performed in triplicate.

Table S2. RNA and DNA sequences used in this study. Related to Methods.
